# A prospective study on the pathogenesis of catheter-associated bacteriuria in critically ill patients

**DOI:** 10.1186/s12866-021-02147-9

**Published:** 2021-03-22

**Authors:** Claire Aumeran, Benoit Mottet-Auselo, Christiane Forestier, Paul-Alain Nana, Claire Hennequin, Frédéric Robin, Bertrand Souweine, Ousmane Traoré, Alexandre Lautrette

**Affiliations:** 1 Infection Control Department, 3IHP, CHU Clermont-Ferrand, 63000 Clermont-Ferrand, France; 2 Université Clermont Auvergne, UMR CNRS 6023 ‘Laboratoire Microorganismes: Génome Environnement (LMGE)’, F-63000 Clermont-Ferrand, France; 3 Service d’Hygiène Hospitalière, Hôpital Gabriel Montpied, 58 Rue Montalembert, 63003 Clermont-Ferrand Cedex 1, France; 4 Bacteriology Department, 3IHP, CHU Clermont-Ferrand, 63000 Clermont-Ferrand, France; 5 Université Clermont Auvergne, UMR INSERM 1071 ‘Laboratoire Microbe intestin inflammation et Susceptibilité de l’Hôte (M2ISH)’, USC INRA2018, F-63000 Clermont-Ferrand, France; 6 Laboratoire associé Résistance des Entérobactéries BLSE/Céphalosporinases, Centre National de Référence Résistance aux Antibiotiques, Clermont-Ferrand, France; 7 Intensive Care Medicine, CHU Clermont-Ferrand, 63000 Clermont-Ferrand, France

**Keywords:** Catheter-associated bacteriuria, Critically ill patients, Pathogenesis

## Abstract

**Background:**

Updating the pathogenesis of catheter-associated bacteriuria (CA-bacteriuria) in the intensive care unit (ICU) is needed to adapt prevention strategies. Our aim was to determine whether the main pathway of CA-bacteriuria in ICU patients was endoluminal or exoluminal.

In a prospective study, quantitative urine cultures were sampled from catheter sampling sites, collector bags and the catheter outer surface near the meatus from days 1 to 15 after catheterization. The endoluminal pathway was CA-bacteriuria (defined as 10^2^ CFU/mL) first in collector bags and then in catheters. The exoluminal pathway was CA-bacteriuria first in catheters, on day 1 in early cases and after day 1 in late cases.

**Results:**

Of 64 included patients, 20 had CA-bacteriuria. Means of catheterization days and incidence density were 6.81 days and 55.2/1000 catheter-days. Of 26 microorganisms identified, 12 (46.2%) were Gram positive cocci, 8 (30.8%) Gram negative bacilli and 6 yeasts*.* Three (11.5%) CA-bacteriuria were endoluminal and 23 (88.5%) exoluminal, of which 10 (38.5%) were early and 13 (50%) late. Molecular comparison confirmed culture findings. A quality audit showed good compliance with guidelines.

**Conclusion:**

The exoluminal pathway of CA-bacteriuria in ICU patients predominated and surprisingly occurred early despite good implementation of guidelines. This finding should be considered in guidelines for prevention of CA-bacteriuria.

**Supplementary Information:**

The online version contains supplementary material available at 10.1186/s12866-021-02147-9.

## Background

With a prevalence of up to 40%, urinary tract infections (UTI) are the first cause of nosocomial infections [1–5]. The presence of a urinary catheter is the main risk factor for nosocomial UTI [2, 3]. A diagnosis of catheter-associated bacteriuria (CA-bacteriuria) is established when no distinction is made between catheter-associated asymptomatic bacteriuria and catheter-associated urinary tract infection (CA-UTI) [1]. CA-bacteriuria is correlated with the duration, mainly 6 days, of catheterization [1, 6]. The risk of CA-UTI increases in the intensive care unit (ICU), where incidence rates range between 3.6 and 14.71 per 1000 urine catheter days [1, 3, 5, 7, 8]. The first step in the pathogenesis of CA-UTI is the endoluminal or exoluminal colonization of the urinary catheter, which is more frequently involved than the blood-borne pathway [1, 6, 9]. Some studies have reported that endoluminal CA-bacteriuria involves exogenous flora originating from the colonization of the collector bag or a breach of the closed system during manipulations of the urinary catheter [10–13]. Exoluminal CA-bacteriuria involves endogenous flora from the urinary meatus. This kind of colonization occurs early during the insertion of the catheter or later as a result of the colonization of the urinary meatus by the digestive flora [11–18]. After adhesion, the microorganisms migrate within a biofilm along the endoluminal and exoluminal sides of the urinary catheter. In 1999, a study reported the predominance of the exoluminal pathway in a non-selected population [9]. No study has explored the pathway mechanisms of CA-bacteriuria solely in critically ill patients. The impact of guidelines on the pathogenesis of pathways for CA-bacteriuria is unknown [1, 2, 4, 19, 20]. Up-to-date knowledge of these pathways is needed to improve the prevention of CA-bacteriuria and hence CA-UTI [1, 2, 4, 19], which is one of the most frequent nosocomial infections in the ICU and is associated with a heavy health burden [1, 2, 19, 21, 22].

The main aim of this prospective study was to explore the pathways (exoluminal vs endoluminal) of CA-bacteriuria in critically ill patients. Secondarily, we investigated the characteristics of patients and microorganisms involved in the infection and performed a quality audit on urinary catheterization.

## Results

**Fig. 1:**
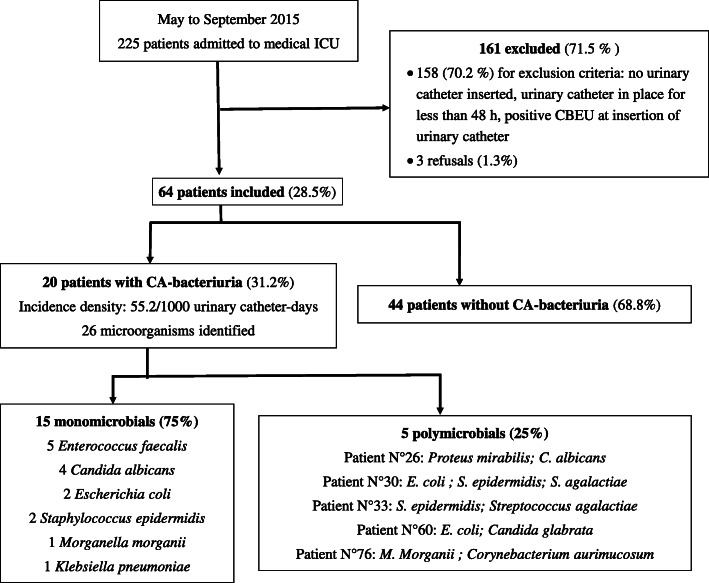
Flow chart of study. CA-bacteriuria: catheter-associated bacteriuria, ICU: Intensive Care Unit, CBEU: cytobacteriological examination of the urine

Of the 225 patients admitted to the ICU (Fig. 1), 64 were included. CA-bacteriuria was identified in 20 patients (31.2%), of whom 15 were monomicrobial and 5 polymicrobial, corresponding to an incidence density of 55.2 per 1000 urinary catheter-days. There was no difference in patient characteristics between those with CA-bacteriuria and those without, except for the sex/ratio and for the number of prescriptions of anti-infective therapy before catheterization or during patient follow-up (Table 1). Most of the urinary catheters were manufactured with 100% silicone (*n* = 54, 84.4%). The mean duration of catheterization was 6.81 ± 0.58 days, with no difference between patients with or without CA-bacteriuria (Table 1). For 39 (60%) patients, the duration of catheterization was less than 6 days. Four patients had CA-UTI, which gave an incidence density of 9.1 per 1000 urinary catheter-days. No bacteremia or fungemia with the same microorganism as CA-bacteriuria was reported (See Supplementary Table 1, Additional File 1).

**Table 1:** Comparison of included patients with and without catheter-associated bacteriuria (CA-bacteriuria)

Variables	Patients		
	CA-bacteriuria (*n* = 20; 31.2%)	No CA-bacteriuria (*n* = 44; 68.8%)	*p. value*
Demographics			
Age (years)^a^	71 ± 12.9	63.7 ± 15.5	*0.06*
Male/Female (number)	8/12	37/7	*0.001*
Medical past history			
Chronic kidney disease (%)	4 (20%)	5 (11.4%)	*0.44*
Diabetes mellitus (%)	3 (15%)	4 (9.1%)	*0.66*
Primary disease			
Acute renal failure (%)	2 (10%)	1 (2.3%)	*0.23*
Cardiac arrest (%)	1 (5%)	3 (6.8%)	*1.00*
Coma (%)	4 (20%)	7 (15.9%)	*0.73*
Postoperative care (%)	1 (5%)	3 (6.8%)	*1.00*
Respiratory failure (%)	2 (10%)	1 (2.3%)	*0.23*
Sepsis (%)	5 (25%)	17 (38.6%)	*0.39*
Shock (%)	4 (20%)	11 (25%)	*0.75*
Traumatism (%)	1 (5%)	1 (2.3%)	*0.53*
ICU Data			
Length of stay (days)^a^	11.3 ± 10.1	11.3 ± 8.8	*0.99*
Admission weight (Kg)^a^	82.9 ± 25.7	76.4 ± 24.7	*0.34*
BMI (kg/m^2^)^a^	30.5 ± 9.5	30.6 ± 30.2	*0.98*
SAPS II score^a^	50.3 ± 29.2	50.3 ± 21.8	*0.69*
Anti-infective therapy^b^			
Prior to urinary catheterization (%)	6 (30%)	34 (77.3%)	*0.0006*
During patient follow-up (%)	10 (50%)	35 (79.5%)	*0.04*
Mean duration (days) ^ab^	6.45 ± 1.72	7.68 ± 1.05	*0.55*
Complications			
Death (%)	1 (5%)	5 (11.4%)	*0.65*
Mechanical ventilation (%)	8 (40%)	22 (50%)	*0.59*
Non-invasive ventilation (%)	10 (50%)	27 (61.4%)	*0.42*
Vasoactive amine (%)	9 (45%)	16 (36.4%)	*0.58*
Acute renal failure (%)	5 (25%)	9 (20.5%)	*0.74*
Dialysis (%)	3 (15%)	6 (13.6%)	*1.00*
Urinary catheterization			
100% silicon catheter (%)	16 (80%)	38 (86.3%)	*0.71*
Silicon-coated-latex catheter (%)	4 (20%)	6 (13.7%)	*0.71*
Mean duration of catheterization (days) ^a^	6.45 ± 0.94	6.98 ± 0.73	*0.89*

*BMI* Body Mass Index, *SAPS II* simplified acute physiology score II

Statistical significance*: P* values of < 0.05

^a^Results expressed as mean ± standard deviation

^b^Not all patients required anti-infective therapy before catheter insertion or during follow-up. In the CA-bacteriuria group, only four patients had a catheter-associated urinary tract infection with the microorganism responsible for bacteriuria. They were treated accordingly to the identified microorganism. The other anti-infective therapies were secondary to another infection

Of the 26 microorganisms identified, 12 (46.2%) were Gram-positive cocci, with a predominance of *E. faecalis* (*n* = 5, 19.2%) and *S. epidermidis* (*n* = 4, 15.4%), 8 (30.8%) were Gram-negative bacilli, with a predominance of *E. coli* (*n* = 4, 15.4%), and 6 (23.1%) were *Candida sp.* (Fig. 1). The list of microorganisms obtained in the urethral flora samples is shown in the Additional files (See Supplementary Table 1, Additional File 1).

**Fig. 2:**
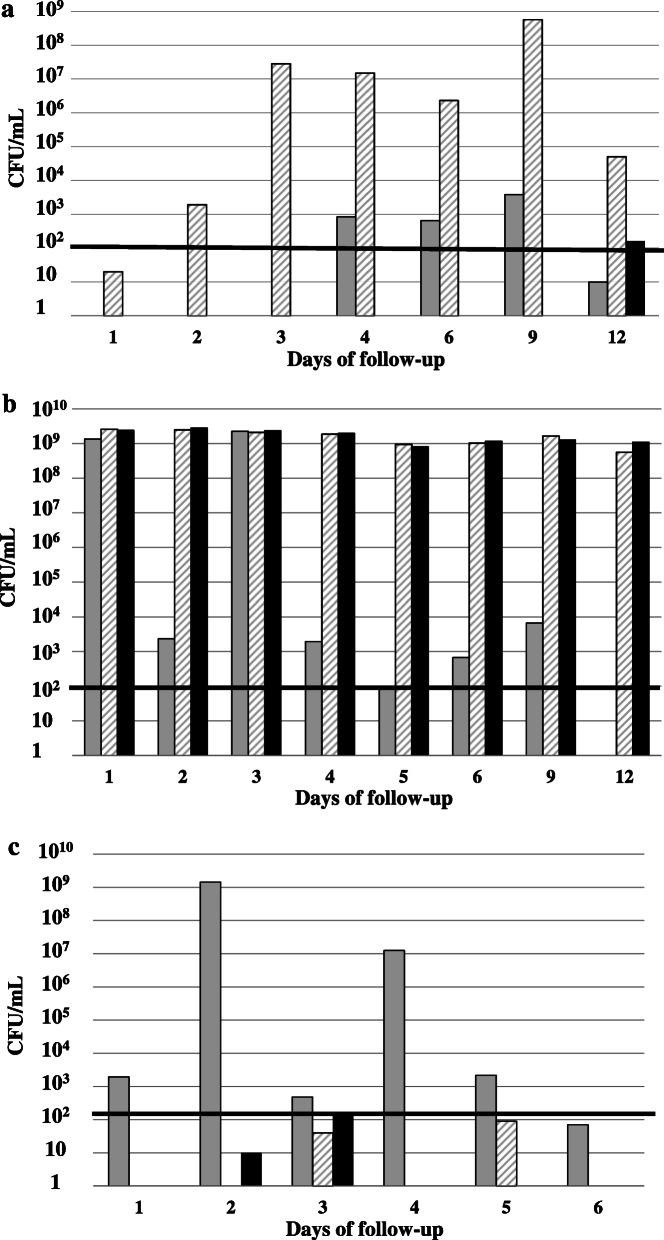
Origin of catheter-associated bacteriuria. Black line: Threshold of detection of 10^2^ CFU/mL that defines catheter-associated bacteriuria. Black bar: Bladder urine; grey bar: outer surface of the urinary catheter near the urinary meatus; hatched bar: urine from the collector bag. Figures 2**a**, **b** and **c** give an example of the dynamics of the occurrence of CA-bacteriuria of each group (**a** endoluminal, **b** early exoluminal, **c** late exoluminal) which reflects the dynamics in the whole group. CA-bacteriuria was defined as a number of microorganisms higher than or equal to 10^2^ CFU/mL in at least one bladder urine sample. The origin of CA-bacteriuria was defined by comparing the dynamics of the occurrence of microorganisms in bladder urine, collector bag urine and urinary catheter swabs. **a** Endoluminal catheter-associated bacteriuria. Identification of microorganisms, first in collector bag urine and then in bladder urine. *Morganella morganii* in patient 76, first in collector bag urine on day 2 and then in bladder urine on day 12. The molecular comparison is given in Additional file 2 (See Supplementary Figures 14 and 16, Additional File 2). **b** Early exoluminal catheter-associated bacteriuria. Identification of microorganisms on the first day of catheterization in bladder urine with or without identification in urine from collector bags. *Klebsiella pneumoniae* in patient 22 on the first day of catheterization in bladder urine. It was also detected from day 1 in the collector bag and on the outer surface of the urinary catheter near the urinary meatus. The molecular comparison is given in Additional file 2 (See Supplementary Figure 17, Additional File 2). **c**. Late exoluminal catheter-associated bacteriuria. Identification of microorganisms after the first day of catheterization in bladder urine without a preliminary identification in urine from collector bags. *Morganella morganii* in patient 56 in bladder urine and collector bag urine on day 3. It was detected from day 1 on the outer surface of the urinary catheter near the urinary meatus. The molecular comparison is given in Additional file 2 (See Supplementary Figures 14 and 15, Additional File 2)

There were 3 (11.5%) endoluminal and 23 (88.5%) exoluminal cases of CA-bacteriuria (*n* = 23, 88.5%), of which 10 (38.5%) were early and 13 (50%) late (Fig. 2a, b and c; see Supplementary Table 1, Additional File 1; see Supplementary Figures 14 to 17, Additional file 2). Four patients had CA-UTI. Patient 22 had early exoluminal *Klebsiella pneumoniae* CA-bacteriuria followed by acute prostatitis treated with cefotaxime. The three other patients were classified as cases of late exoluminal CA-bacteriuria. Patient 26 was infected by *Proteus mirabilis* and treated with cefotaxime, cefepime and then meropenem. Patient 44 was infected by *Escherichia coli* and treated with cefotaxime. Patient 75 was infected by *Escherichia coli* and treated with nitrofurantoin (See Supplementary Table 1, Additional File 1). No difference was observed when the endoluminal and exoluminal groups of CA-bacteriuria were compared, whether for major risk factors (duration of catheterization, chronic kidney disease, and diabetes mellitus), outcomes (death, acute renal failure, dialysis, mechanical ventilation invasive or not, and use of amines), gender, age or the microorganisms identified. No difference was found either when early and late exoluminal groups of CA-bacteriuria were compared. Detailed data are presented in Supplementary Table 2, Additional file 1. Molecular comparison confirmed culture findings in all but one patient, in whom early exoluminal developed into endoluminal CA-bacteriuria (*S. epidermidis*, patient 33). Since *Staphylococcus epidermidis* and *Candida sp.* are not common uropathogens, the results of their molecular comparisons are given in Additional file 2 (See Supplementary Figures 1 to 13, Additional File 2). In 16 cases (69.5%), comparison with microorganisms identified on swabs confirmed early exoluminal (*n* = 7/10) and late exoluminal CA-bacteriuria (*n* = 9/13).

The quality audit was performed without prior notice by the same intensivist in all observations. The quality audit on urinary catheterization showed that there were no problems in following international catheterization guidelines [2, 4, 19]. All hand hygiene procedures were complied with but 50% were performed with alcohol-based products (Table 2).

**Table 2:** Quality audit of the insertion of a urinary catheter

Questions	% of answers in accordance with recommendations
Knowledge of protocol	100% (10/10)
Two people performing urinary catheterization	80% (8/10)
Genital cleaning	100% (10/10)
Sterile single-dose plain soap	70% (7/10)
Non-sterile plain soap	10% (1/10)
Chlorhexidine scrub	20% (2/10)
Hand hygiene before genital cleaning	100% (10/10)
**Alcohol-based hand rubbing**	**50% (5/10)**
Plain soap handwashing	20% (2/10)
Antiseptic soap handwashing	30% (3/10)
Use of vinyl gloves	100% (10/10)
Hand hygiene after genital cleaning	80% (8/10)
**Alcohol-based hand rubbing**	**50% (4/8)**
Plain soap handwashing	25% (2/8)
Antiseptic soap handwashing	25% (2/8)
Installer: mask, hygiene cap, disposable apron	100% (10/10)
Antisepsis of urinary meatus with sterile gloves	100% (10/10)
Dakin solution	80% (8/10)
Chlorexhidine 0.05% (sterile single dose)	20% (2/10)
Hand hygiene after sterile glove removal	100% (10/10)
**Alcohol-based hand rubbing**	**50% (5/10)**
Plain soap handwashing	20% (2/10)
Antiseptic soap handwashing	20% (2/10)
Sterile urinary catheterization	100% (10/10)
Hand hygiene after catheterization	90% (9/10)
**Alcohol-based hand rubbing**	**56% (5/9)**
Plain soap handwashing	22% (2/9)
Antiseptic soap handwashing	22% (2/9)
Securing the collector bag in a sloping position	100% (10/10)
Traceability = date of catheterization	
On collector bag	100% (10/10)
In patient file	90% (9/10)

10 observations were made by one investigator of 10 consecutive catheterizations. The audit grid was established in accordance with national recommendations [4]

## Discussion

This study investigated the pathways of CA-bacteriuria. The exoluminal pathway was predominant and accounted for more than one third of cases of early exoluminal CA-bacteriuria. This result was not related to the quality of urinary catheterization.

CA-bacteriuria occurred in one third of patients admitted to the Medical ICU without any effect on catheterization duration in contrast what is expected with CA-UTI. The proportion of CA-bacteriuria cases was consistent with that in other ICU studies [23, 24]. We observed a greater number of cases of exoluminal CA-bacteriuria (88.5%) and a low rate of endoluminal CA-bacteriuria (11.5%). In our study, the main pathway was exoluminal and occurred at a higher rate than in the study of Tambyah et al., in which 46, 23.2 and 30.8% of CA-bacteriuria cases were exoluminal, endoluminal and indeterminate CA-bacteriuria, respectively [9]. These authors reported a 10% rate of disconnections between urinary catheters and collector bags, which is a risk factor for endoluminal CA-bacteriuria. During the 4 months of our study, only two disconnections (3%) in 64 included patients were observed, which could explain the very low proportion of endoluminal CA-bacteriuria cases. These findings suggest that by concentrating attention on preventing disconnection the guidelines lead to a large reduction in the endoluminal pathway [1, 2]. Another explanation could be the increasing use of 100% silicon urinary catheters, which delay the obstruction caused by biofilm encrustation [27].

Tambyah et al. observed 12.4% (*n* = 31) of early exoluminal CA-bacteriuria cases among 250 identified microorganisms [9] as against 38.5% in our study. Early exoluminal colonization could be related to a defect in the aseptic procedure during insertion of the catheter. To assess this assumption, we performed a quality audit on catheter insertion in accordance with the most recent guidelines [2, 4, 19]. The only flaw observed was a low observance of hydro-alcoholic hand rubbing (approximately 50%) during the different stages of insertion where alcohol-based products are recommended. This failure was not a major infringement of the guidelines but could have been involved in early exoluminal CA-bacteriuria. However, the microorganisms of early exoluminal colonization in our study did not come only from the skin flora. Another cause of early exoluminal CA-bacteriuria could be the presence of microorganisms in the final centimeters of the urethra carried away during catheterization. In our study, 69.5% of the microorganisms of exoluminal CA-bacteriuria were also identified on samples of urinary meatus. This is consistent with other reports which showed that in 75% of cases the microorganisms of exoluminal bacteriuria preexisted in the urethral flora [15, 16] and that a positive culture of urinary meatus increased the incidence of CA-bacteriuria [17, 18, 28] However, meatal care does not result in a reduction in CA-UTI or urethral colonization [1, 20]. Before insertion, there is a physical inability to access the inside of the urethra during antisepsis [9]. It is likely that meatal care does not completely eliminate the microbial flora present in the last few centimeters of the urethra. In addition, the presence of diarrhea and the catheter maintenance environment could affect late exoluminal CA-bacteriuria.

After insertion, the persistence of CA-bacteriuria could be related to the formation of biofilm at the interface of the catheter and the urethra, to repeated catheter manipulations or to inadequate residual activity of the antiseptic [1]. Consequently, the strict implementation of guidelines by health workers does not avoid exoluminal CA-bacteriuria.

The first limitation of our study is the low number of patients included. Because of the lack of data available in the literature on this subject, no calculation of population size has yet been made. However, this was compensated by the large number of samples (*n* = 1008 with a maximum of 27 samples for 15 days), which is indicative of a good follow-up of each patient. We do not exclude the possibility that, owing to a lack of statistical power, rare events could not be highlighted. A second limitation was the viable non-cultivable bacteria that we were unable to isolate. To reduce this bias, it would have been necessary to remove the urinary catheter and culture its end or to carry out a molecular analysis with 16S PCR. However, as we were performing a real-life observational study, we used the usual microbial analysis techniques. However, this is the first report to confirm microbial results by molecular comparison of the same microorganisms isolated. A final limitation was the choice of a threshold of 10^2^ CFU/mL in bladder urine to define CA-bacteriuria: in French and American guidelines, it is set at 10^3^ CFU/ml [1, 4]. This could have artificially increased the detection of CA-bacteriuria. However, our aim was earlier detection of bacteriuria. The change in threshold was possible because standardization of the microbiological technique allowed a detection threshold of 10 CFU/m. We do not rule out an impact of anti-infective therapy on the detection threshold. However, there was no difference in mean duration of anti-infective therapy between the two groups. In addition, it is established that in patients with a urinary catheter not receiving antimicrobial therapy, bacteriuria or candiduria ≥10^2^ CFU/ml will increase to > 10^5^ CFU/mL in 1 to 3 days if the urinary catheter remains in place [1, 4].

## Conclusions

This prospective observational study assessed the occurrence of CA-bacteriuria in a medical ICU. The exoluminal pathway was predominant, and even when guidelines were fully complied with, 38.5% of cases of CA-bacteriuria were early exoluminal. Two factors could explain these findings: the inability to remove the microorganisms from the final centimeters of the urethra during meatal care, and the design of catheter materials. To explore the first hypothesis, a better knowledge of urethral and perineum microbiota is needed. Measures could then be taken to act on the balance of these microbiota to prevent their adhesion to urinary catheters. To explore the second hypothesis, the mechanisms of CA-bacteriuria and biofilm formation on urinary catheters need to be elucidated to guide research into new and safe devices. It would be interesting to study bacterial adhesion to different kinds of urinary catheters according to variations in the environment such as the composition or pH of urine. This could lead to the development of new devices or to medical modification of the composition of urine. The prevention of CA-bacteriuria and, even more importantly, of CA-UTI is a challenge for the medical community, which should now develop interdisciplinary innovation projects.

## Methods

### Population study

We conducted a four-month (from May to September, 2015) observational and prospective clinical study in a 16-bed medical intensive care unit of the University Hospital of Clermont-Ferrand, France. The study was approved by the regional *ethics committee of South-East France* 6 (Comité de Protection des Personnes Sud-Est 6, reference # N°2015/CE 59 - IRB00008526). Patients were informed of the nature and purpose of the study as requested in French guidelines. Informed consent is obtained from all participants and in case of participants who are dead now informed consent is obtained from their legal guardian. Inclusion criteria were adult patients with urinary catheter inserted for a duration of more than 48 h. Catheterization was performed only in the ICU. Exclusion criteria were no urinary catheter or catheter inserted for a duration of less than 48 h, identification of a microorganism by cytobacteriological examination of the urine (CBEU) on the day the catheter was inserted (day-0), pregnant or breastfeeding women and subjects protected by law. Patient care, choice and management of the urinary catheter were left to the discretion of the healthcare team. There was continuous monitoring of disconnections during follow-up by the ICU care team. Infections were defined according to American and French guidelines [1, 2, 4].

### Sampling

Two investigators from the infection control team performed samplings. Patient follow-up did not exceed 15 days depending on the duration of catheterization and hospital stay. Samples were taken daily from the first to the sixth day of catheterization and then on the ninth, twelfth and fifteenth days. Each time, three samples were taken **(i)** in bladder urine at the urinary catheter sampling site according to national recommendations using a needle or suitable adapter [4] **(ii)** in urine from the collector bag after disinfection of the drain end-piece with chlorhexidine-alcohol 0.5% and **(iii)** in swabs with transport media (Transystem®, COPAN©, Brescia, Italy) of the outer surface of the urinary catheter near the urinary meatus. All samples were collected in CBEU tubes (BD Vacutainer®, BD Diagnostics©, Le Pont de Claix, France).

### Microbiological analysis

Each sample was streaked on two agar plates for Gram-negative bacilli (Drigalski agar, bioMerieux©, Craponne, France) and for Gram-positive bacteria and yeasts (Columbia CAP Agar, Oxoid©, Basingstoke, United Kingdom). Plating was standardized (easySpiral Dilute®, Interscience©, Saint Nom la Brétèche, France) and 100 μL were inoculated on agar to obtain a threshold detection of 10 colony-forming units (CFU)/ml. Each agar plate was incubated at 37 °C for 48 h and the bacterial count was automatized (Scan® 500, Interscience©, Saint Nom la Brétèche, France). Results were expressed quantitatively for urine samples and semi-quantitatively for swab samples because they were drained in 1000 μL of saline solution before being streaked. When the CFU count from the bladder urine sample was higher than or equal to 10^2^ CFU/mL, all strains isolated from the patient were identified by MALDI TOF mass spectrometry (Matrix-Assisted Laser Desorption/Ionisation time-of-flight, VITEK® MS, bioMerieux©, Craponne, France). For each patient, clonal relatedness was determined either by pulsed-field gel electrophoresis (PFGE) or by ERIC2-PCR on isolates identified during follow-up. PFGE was performed with the GenePath System (Bio-Rad Laboratories, Marnes la Coquette, France) according to the manufacturer’s instructions for *Staphylococcus epidermidis, Enterococcus faecalis, Candida albicans* and *Candida glabrata.* Isolates were grown in 5 mL of Trypticase soy broth at 37 °C for 16 to 20 h. Following digestion with the restriction enzymes SmaI or BssHII (New England Biolabs, Ipswich, MA, USA), DNA fragments were separated using the GenePath instrument. SmaI was used for *Staphylococcus epidermidis* and *Enterococcus faecalis,* and BssHII for *Candida albicans* and *Candida glabrata.* The run conditions were 6 V/cm, 22 h, 120° angle, linear ramp, initial switch 2.2 s, final switch 54.2 s. DNA banding patterns were interpreted according to Tenover et al. [29]. Isolates were considered to be closely related if the PFGE patterns differed by three or fewer bands. ERIC2-PCR was performed according to Dumarche et al. for *Escherichia coli*, *Klebsiella pneumoniae*, *Morganella morganii*, and *Proteus mirabilis*. No molecular comparison was performed for *Corynebacterium aurimucousum* and *Streptococcus agalactiae* because these strains are rarely involved in urinary tract infections.

### Establishing the first localization of catheter-associated bacteriuria (CA-bacteriuria)

The first localization of CA-bacteriuria was defined by observing the dynamics of the occurrence of microorganisms in the bladder urine and collector bags. CA-bacteriuria was defined as a number of microorganisms higher than or equal to 10^2^ CFU/mL in at least one bladder urine sample collected at the urinary catheter collection site. As previously reported [9], the CA-bacteriuria pathway was identified by comparing the dynamics of the occurrence of microorganisms on the three samples (bladder urine, collector bag urine, urinary catheter swabs). Endoluminal CA-bacteriuria was defined by the identification of the same microorganism first in collector bag urine and then in bladder urine. Early exoluminal CA-bacteriuria was defined by the identification of the same microorganism on the first day of catheterization in bladder urine with or without identification in urine from the collector bag. Late exoluminal CA-bacteriuria was defined by the identification of the same microorganism after the first day of catheterization in bladder urine without preliminary identification in urine from the collector bag. A molecular comparison of the strains was then performed that confirmed the culture findings. In some cases, molecular comparison with microorganisms identified on swabs helped to confirm exoluminal CA-bacteriuria.

### Quality audit of urinary catheter insertion

We carried out a quality audit on urinary catheter insertion according to our hospital protocol, which is based on the international guidelines [2, 4, 19]. One intensivist investigator from the medical ICU, who was trained by the infection control team, observed the insertion of 10 consecutive urinary catheters. The audit grid followed the insertion protocol and included the knowledge of protocol, the number of people performing the insertion, the kind of hand hygiene and the personal protective equipment used, the kind of meatal cleansing and antisepsis of the urinary meatus, the respect of asepsis, positioning of the collector bag and traceability of catheter insertion in the medical file.

### Statistical analyses

Patients with and without CA-bacteriuria were compared with Student test for quantitative data, and a Fisher or CHI2 test for qualitative data. The occurrence of each microorganism was analyzed as an independent case of CA-bacteriuria. Categorical data were expressed as numbers and percentages, and quantitative parameters as mean ± standard-deviation. *P* values of < 0.05 were considered to indicate statistical significance. Analyses were performed with SAS® software (SAS Institute Inc.). SAS and all other SAS Institute Inc. product or service names are registered trademarks or trademarks of SAS Institute Inc. in the USA and other countries. ® indicates USA registration.

## Supplementary Information

**Additional file 1: Supplementary Table 1.** Dynamics of catheter-associated bacteriuria (CAB), list of microorganisms identified in the urinary meatus, and infected patients. **Supplementary Table 2**. Comparison of the 26 catheter-associated bacteriuria groups, endoluminal versus exoluminal, including a subgroup comparison of early versus late exoluminal catheter-associated bacteriuria.

**Additional file 2: Supplementary Figures 1 to 13.** Molecular comparisons of *Staphylococcus epidermidis, Candida sp*. **Supplementary Figures 14 to 17.** Molecular comparisons corresponding to Fig. 2a, b and c.

## Abbreviations

CA-UTI: Catheter-associated urinary tract infection
CA-Bacteriuria: Catheter-associated bacteriuria
ICU: Intensive care unit
