# Supplementary material for: A prospective study on the pathogenesis of catheter-associated bacteriuria in critically ill patients
Source: BMC Microbiol. 2021 Mar 22;21:86. doi: 10.1186/s12866-021-02147-9 (PMC7983228; doi:10.1186/s12866-021-02147-9)
Supplement: Supplementary file 1 — Additional file 1: Supplementary Table 1. Dynamics of catheter-associated bacteriuria (CAB), list of microorganisms identified in the urinary meatus, and infected patients. Supplementary Table 2. Comparison of the 26 catheter-associated bacteriuria groups, endoluminal versus exoluminal, including a subgroup comparison of early versus late exoluminal catheter-associated bacteriuria. [file 12866_2021_2147_MOESM1_ESM.docx]

A prospective study on the pathogenesis of catheter-associated bacteriuria in critically ill patients

Claire Aumeran,^1,2 ✉^; Benoit Mottet-Auselo,^1^; Christiane Forestier,^2^; Paul-Alain Nana,^2^; Claire Hennequin,^2,3^; Frédéric Robin,^3,4,5^; Bertrand Souweine,^2,6^; Ousmane Traoré,^1,2^; Alexandre Lautrette, ^2,6^

^1^ Infection Control Department, 3IHP, CHU Clermont-Ferrand, 63000, Clermont-Ferrand, France

^2^ Université Clermont Auvergne, UMR CNRS 6023 ‘Laboratoire Microorganismes: Génome Environnement (LMGE)’, F-63000, Clermont-Ferrand, France

^3^ Bacteriology Department, 3IHP, CHU Clermont-Ferrand, 63000, Clermont-Ferrand, France

^4^ Université Clermont Auvergne, UMR INSERM 1071 ‘Laboratoire Microbe intestin inflammation et Susceptibilité de l'Hôte (M2ISH)’, USC INRA2018, F-63000, Clermont-Ferrand, France

^5^ Laboratoire associé Résistance des Entérobactéries BLSE/Céphalosporinases, Centre National de Référence Résistance aux Antibiotiques, Clermont-Ferrand, France.

^6^ Intensive Care Medicine, CHU Clermont-Ferrand, 63000, Clermont-Ferrand, France

^✉^**Corresponding author**: Dr Claire Aumeran (MD-PhD). email: [caumeran@chu-clermontferrand.fr](mailto:caumeran@chu-clermontferrand.fr).

**Additonal file 1: Supplementary Table 1 and supplementary Table 2**

**Supplementary Table 1:** Dynamics of catheter-associated bacteriuria (CAB), list of microorganisms identified in the urinary meatus, and infected patients

| Patient  Sex | CAB origin | Bladder urine MO  (Day of occurrence) | Collector bag MO  (Day of occurrence) | Urinary meatus  (List of microorganisms) | Infection |
| --- | --- | --- | --- | --- | --- |
| 30  F | Endoluminal | *E. coli*  (6) | *E. coli*  (2) | *Escherichia coli*  *Enterobacter cloacae*  *Staphylococcus epidermidis*  *Streptococcus agalactiae*  *Enterococcus faecalis* | / |
| 33  M | Endoluminal | *S. epidermidis*  (3) | *S. epidermidis*  (1) | *Escherichia coli*  *Streptococcus agalactiae*  *Staphylococcus lugdunensis*  *Candida albicans* | / |
| 76  F | Endoluminal | *M. morganii*  (12) | *M. morganii*  (1) | *Morganella morganii**  *Corynebacterium aurimucosum*  *Escherichia coli* | / |
| 23  F | Early exoluminal | *C. albicans*  (1) | *C. albicans*  (1) | *Candida albicans*  *Pseudomonas aeruginosa* | / |
| 34  M | Early exoluminal | *C. albicans*  (1) | / | *Candida albicans*  *Staphylococcus capitis* | / |
| 46  M | Early exoluminal | *C. albicans*  (1) | *C. albicans*  (1) | *Candida albicans*  *Staphylococcus capitis* | / |
| 60  F | Early exoluminal | *C. glabrata*  (1) | *C. glabrata*  (1) | *Candida glabrata*  *Escherichia coli*  *Pseudomonas aeruginosa*  *Proteus vulgaris*  *Lactobacillus gasseri* | / |
| 76  F | Early exoluminal | *C. aurimucosum*  (1) | *C. aurimucosum*  (1) | *Corynebacterium aurimucosum*  *Morganella morganii*  *Escherichia coli* | / |
| 19  F | Early exoluminal | *E. faecalis*  (1) | *E. faecalis*  (1) | *Enterococcus faecalis*  *Escherichia coli*  *Streptococcus anginosus*  *Candida krusei* | / |
| 59  F | Early exoluminal | *E. faecalis*  (1) | *E. faecalis*  (1) | *Enterococcus faecalis**  *Staphylococcus capitis* | / |
| 22  M | Early exoluminal | *K. pneumoniae*  (1) | *K. pneumoniae*  (1) | *Klebsiella pneumoniae*  *Proteus mirabilis*  *Serratia marcescens Staphylococcus epidermidis*  *Enterococcus faecalis*  *Steptococcus mitis/oralis* | Acute prostatitis  *K. pneumoniae*  Cefotaxime |
| 30  F | Early exoluminal | *S. agalactiae*  (1) | *S. agalactiae*  (1) | *Streptococcus agalactiae*  *Staphylococcus epidermidis*  *Enterococcus faecalis Escherichia coli*  *Enterobacter cloacae* | / |
| 33  M | Early exoluminal | *S. agalactiae*  (1) | *S. agalactiae*  (1) | *Streptococcus agalactiae*  *Staphylococcus lugdunensis*  *Escherichia coli*  *Candida albicans* | / |
| 26  M | Late exoluminal | *C. albicans*  (5) | *C. albicans*  (6) | *Candida albicans*  *Staphylococcus lugdunensis*  *Staphylococcus epidermidis*  *Proteus mirabilis* | / |
| 51  F | Late exoluminal | *C. albicans*  (2) | *C. albicans*  (2) | *C. albicans*  *Enterococcus faecalis Staphylococcus lugdunensis* | / |
| 44  F | Late exoluminal | *E. coli*  (12) | *E. coli*  (12) | *Escherichia coli*  *Klebsiella pneumonia*  *Staphylococcus epidermidis*  *Enterococcus faecalis*  *Enterococcus faecium*  *Pediococcus acidilactici*  *Micrococcus luteus* | CA-UTI  *E.coli*  Amoxicillin-clavulaic acid  Cefotaxime |
| 60  F | Late exoluminal | *E. coli*  (4) | *E. coli*  (4) | *Escherichia coli*  *Candida glabrata*  *Pseudomonas aeruginosa*  *Proteus vulgaris*  *Lactobacillus gasseri* | / |
| 75 F | Late exoluminal | *E. coli*  (2) | *E. coli*  (2) | *Escherichia coli*  *Staphylococcus capitis*  *Enterococcus faecalis Pseudomonas aeruginosa* | CA-UTI  *E. coli*  Nitrofurantoin |
| 24 F | Late exoluminal | *E. faecalis*  (6) | / | *Enterococcus faecalis*  *Enterococcus avium*  *Escherichia coli*  *Candida glabrata* | / |
| 54 F | Late exoluminal | *E. faecalis*  (4) | *E. faecalis*  (4) | *Enterococcus faecalis*  *Staphylococcus epidermidis*  *Staphylococcus hominis*  *Klebsiella pneumoniae*  *Prevotella buccalis*  *Candida sp.* | / |
| 73  F | Late exoluminal | *E. faecalis*  (9) | *E. faecalis*  (9) | *Enterococcus faecalis*  *Enterococcus faecium* | / |
| 56  M | Late exoluminal | *M. morganii*  (3) | *M. morganii*  (3) | *Morganella morganii*  *Staphylococcus haemolyticus*  *Staphylococcus epidermidis*  *Staphylococcus aureus*  *Enterococcus faecalis* | / |
| 26  M | Late exoluminal | *P. mirabilis*  (12) | / | *Proteus mirabilis*  *Staphylococcus epidermidis*  *Staphylococcus lugdunensis*  *Candida albicans* | CA-UTI  *P. mirabilis*  Cefotaxime  Cefepime  Meropenem |
| 11  M | Late exoluminal | *S. epidermidis*  (3) | *S. epidermidis*  (3) | *Staphylococcus epidermidis**  *Staphylococcus haemolyticus*  *Staphylococcus aureus*  *Staphylococcus capitis*  *Enterococcus faecalis* | / |
| 30  F | Late exoluminal | *S. epidermidis*  (3) | *S. epidermidis*  (3) | *Staphylococcus epidermidis*  *Enterococcus faecalis*  *Streptococcus agalactiae*  *Escherichia coli*  *Enterobacter cloacae* | / |
| 66  M | Late exoluminal | *S. epidermidis*  (3) | / | *Staphylococcus epidermidis**  *Staphylococcus aureus* | / |

The median time of occurrence was 6 days (3 to 12 days) for endoluminal CA-bacteriuria and 4 days (2 to 12 days) for late exoluminal CA-bacteriuria

MO: microorganism; CA-UTI: catheter-associated urinary tract infection

* According to molecular comparison, this strain is different from the strain responsible for CA-bacteriuria

**Supplementary Table 2**: Comparison of the 26 catheter-associated bacteriuria groups, endoluminal *versus* exoluminal, including a subgroup comparison of early *versus* late exoluminal catheter-associated bacteriuria

| Variables | Endoluminal | Exoluminal | | | *p. value* | |
| --- | --- | --- | --- | --- | --- | --- |
|  | (n=3; 11.5%) | Total  (n=23; 88.5%) | Early  (n=10; 38.5%) | Late  (n=13; 50%) | *Endo vs Exo* | *Early vs Late* |
| Demographics |  |  |  |  |  |  |
| Age (years)^a^ | 77.7 ± 9.6 | 74.8 ± 2.6 | 77.5 ± 3.7 | 72.8 ± 3.8 | *0.79* | *0.38* |
| Male/Female (number) | 1/2 | 9/14 | 4/6 | 5/8 | *1.00* | *0.70* |
| Medical past history |  |  |  |  |  |  |
| Chronic kidney disease | 0 | 6 | 4 | 2 | *1.00* | *0.34* |
| Diabetes mellitus | 1 | 7 | 5 | 2 | *1.00* | *0.17* |
| Primary disease |  |  |  |  |  |  |
| Acute renal failure | 0 | 2 | 1 | 1 | *1.00* | *1.00* |
| Cardiac arrest | 0 | 1 | 0 | 1 | *1.00* | *1.00* |
| Coma | 2 | 6 | 4 | 2 | *0.21* | *0.34* |
| Postoperative care | 0 | 1 | 0 | 1 | *1.00* | *1.00* |
| Respiratory failure | 0 | 2 | 1 | 1 | *1.00* | *1.00* |
| Sepsis | 1 | 5 | 3 | 2 | *1.00* | *0.61* |
| Shock | 0 | 5 | 1 | 4 | *1.00* | *0.34* |
| Traumatism | 0 | 1 | 0 | 1 | *1.00* | *1.00* |
| ICU Data |  |  |  |  |  |  |
| Length of stay (days)^a^ | 13.6 ± 6.7 | 12.5 ± 2.4 | 9.1 ± 2.6 | 15.1 ± 3.7 | *0.88* | *0.19* |
| Admission weight (Kg)^a^ | 61.8 ± 6.7 | 83.1 ± 5.2 | 79.9 ± 8.6 | 85.5 ± 6.7 | *0.06* | *0.61* |
| BMI (kg/m²)^a^ | 22.3 ± 2.9 | 30.3 ± 1.9 | 29.4 ± 3.5 | 31 ± 2.1 | *0.09* | *0.70* |
| SAPS II score^a^ | 31.5 ± 8.6 | 52.1 ± 6.2 | 51.0 ± 8.1 | 52.9 ± 9.4 | *0.24* | *0.88* |
| Complications |  |  |  |  |  |  |
| Death | 0 | 1 | 1 | 0 | *1.00* | *0.44* |
| Mechanical ventilation | 1 | 9 | 3 | 6 | *1.00* | *0.67* |
| Non-invasive ventilation | 0 | 10 | 3 | 7 | *0.26* | *0.40* |
| Vasoactive amine | 1 | 12 | 4 | 8 | *1.00* | *0.41* |
| Acute renal failure | 1 | 8 | 4 | 4 | *1.00* | *0.68* |
| Dialysis | 0 | 4 | 2 | 2 | *1.00* | *1.00* |
| Microorganisms |  |  |  |  |  |  |
| *E. coli* | 1 | 3 | 0 | 3 | *0.40* | *0.22* |
| *K. pneumoniae* | 0 | 1 | 1 | 0 | *1.00* | *0.44* |
| *M. morganii* | 1 | 1 | 0 | 1 | *0.22* | *1.00* |
| *P. mirabilis* | 0 | 1 | 0 | 1 | *1.00* | *1.00* |
| *S. epidermidis* | 1 | 3 | 0 | 3 | *0.40* | *0.22* |
| *E. faecalis* | 0 | 5 | 2 | 3 | *1.00* | *1.00* |
| *S. agalactiae* | 0 | 2 | 2 | 0 | *1.00* | *0.17* |
| *C. albicans* | 0 | 5 | 3 | 2 | *1.00* | *0.61* |
| *C. glabrata* | 0 | 1 | 1 | 0 | *1.00* | *0.44* |
| *C. aurimucosum* | 0 | 1 | 1 | 0 | *1.00* | *0.44* |
| Time occurrence (days) ^b^ | 6 (3-12) | 2 (1-12) | 1 | 4 (2-12) | *0.09* | *2.80* |
| Urinary catheterization |  |  |  |  |  |  |
| Mean duration (days) ^a^ | 9.0 ± 3.0 | 6.9 ± 0.8 | 5.9 ± 1.4 | 7.7 ± 1.1 | *0.56* | *0.32* |

There were 26 occurrences of CA-bacteriuria in 20 patients. Four patients had more than one episode of CA-bacteriuria that were of different origins: patient 26 (*Candida albicans* late exoluminal and *Proteus mirabilis* late exoluminal), patient 30 (*Escherichia coli* endoluminal, *Streptoccoccus agalactiae* early exoluminal and *Staphylococcus* *epidermidis* late exoluminal), patient 33 (*Staphylococus epidermidis* endoluminal and *Streptococcus agalactiae* early exoluminal), patient 60 (*Candida glabrata* early exoluminal and *Esherichia coli* late exoluminal), and patient 76 (*Morganella morganii* endoluminal and *Corynebacterium aurimucosum* early exoluminal).

^a^ Results expressed as mean ± standard deviation. ^b^ Results expressed as median (min-max)

BMI: Body Mass Index. SAPS II: simplified acute physiology score II.

Statistical significance*: P* values of < 0.05
