# Supplementary material for: A prospective study on the pathogenesis of catheter-associated bacteriuria in critically ill patients
Source: BMC Microbiol. 2021 Mar 22;21:86. doi: 10.1186/s12866-021-02147-9 (PMC7983228; doi:10.1186/s12866-021-02147-9)
Supplement: Supplementary file 2 — Additional file 2: Supplementary Figures 1 to 13. Molecular comparisons of Staphylococcus epidermidis, Candida sp. Supplementary Figures 14 to 17. Molecular comparisons corresponding to Fig. 2a, b and c. [file 12866_2021_2147_MOESM2_ESM.pptx]

## Slide 1
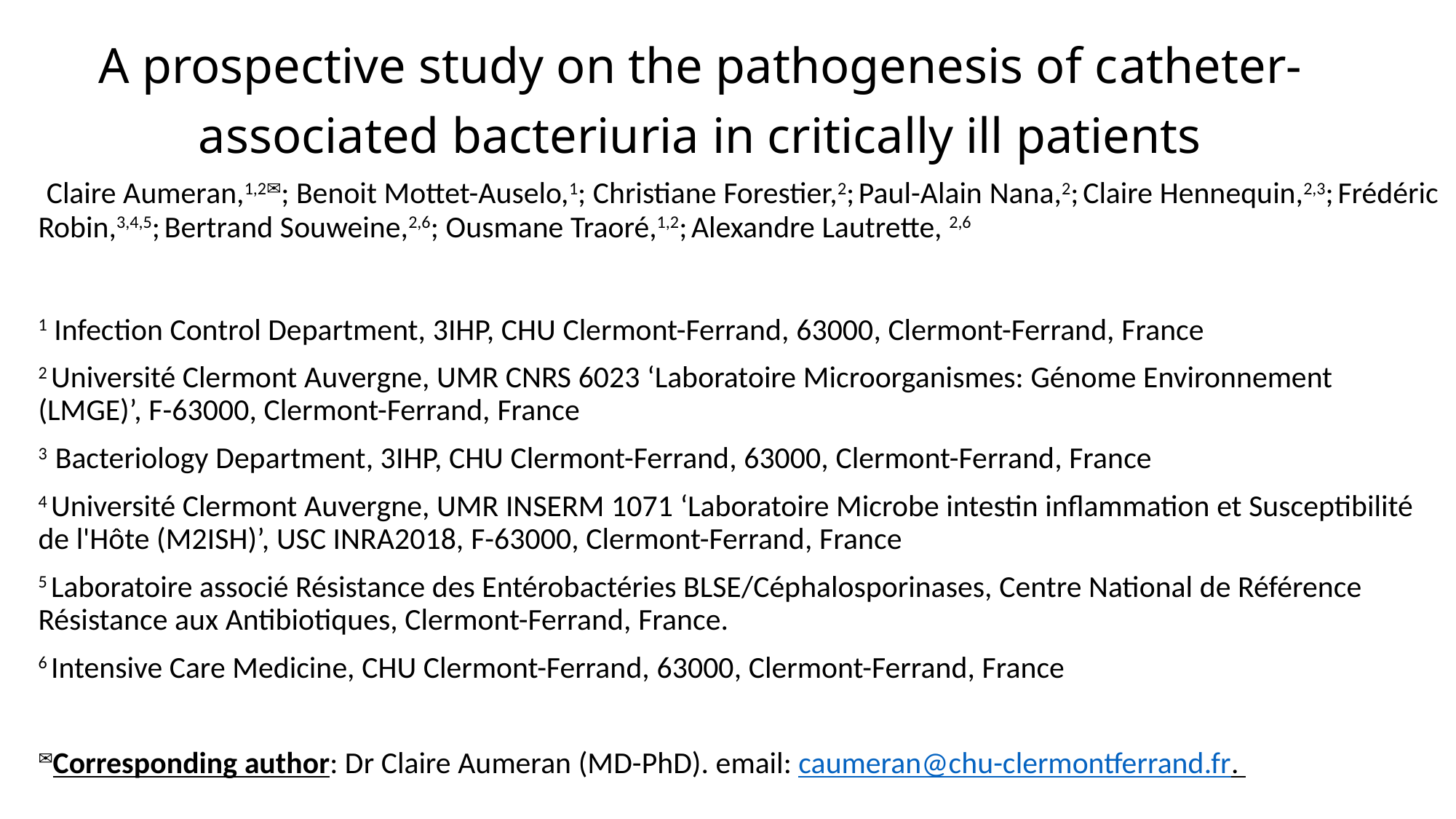

# A prospective study on the pathogenesis of catheter-associated bacteriuria in critically ill patients
 Claire Aumeran,1,2✉; Benoit Mottet-Auselo,1; Christiane Forestier,2; Paul-Alain Nana,2; Claire Hennequin,2,3; Frédéric Robin,3,4,5; Bertrand Souweine,2,6; Ousmane Traoré,1,2; Alexandre Lautrette, 2,6
1 Infection Control Department, 3IHP, CHU Clermont-Ferrand, 63000, Clermont-Ferrand, France
2 Université Clermont Auvergne, UMR CNRS 6023 ‘Laboratoire Microorganismes: Génome Environnement (LMGE)’, F-63000, Clermont-Ferrand, France
3 Bacteriology Department, 3IHP, CHU Clermont-Ferrand, 63000, Clermont-Ferrand, France
4 Université Clermont Auvergne, UMR INSERM 1071 ‘Laboratoire Microbe intestin inflammation et Susceptibilité de l'Hôte (M2ISH)’, USC INRA2018, F-63000, Clermont-Ferrand, France
5 Laboratoire associé Résistance des Entérobactéries BLSE/Céphalosporinases, Centre National de Référence Résistance aux Antibiotiques, Clermont-Ferrand, France.
6 Intensive Care Medicine, CHU Clermont-Ferrand, 63000, Clermont-Ferrand, France
✉Corresponding author: Dr Claire Aumeran (MD-PhD). email: caumeran@chu-clermontferrand.fr.

## Slide 2
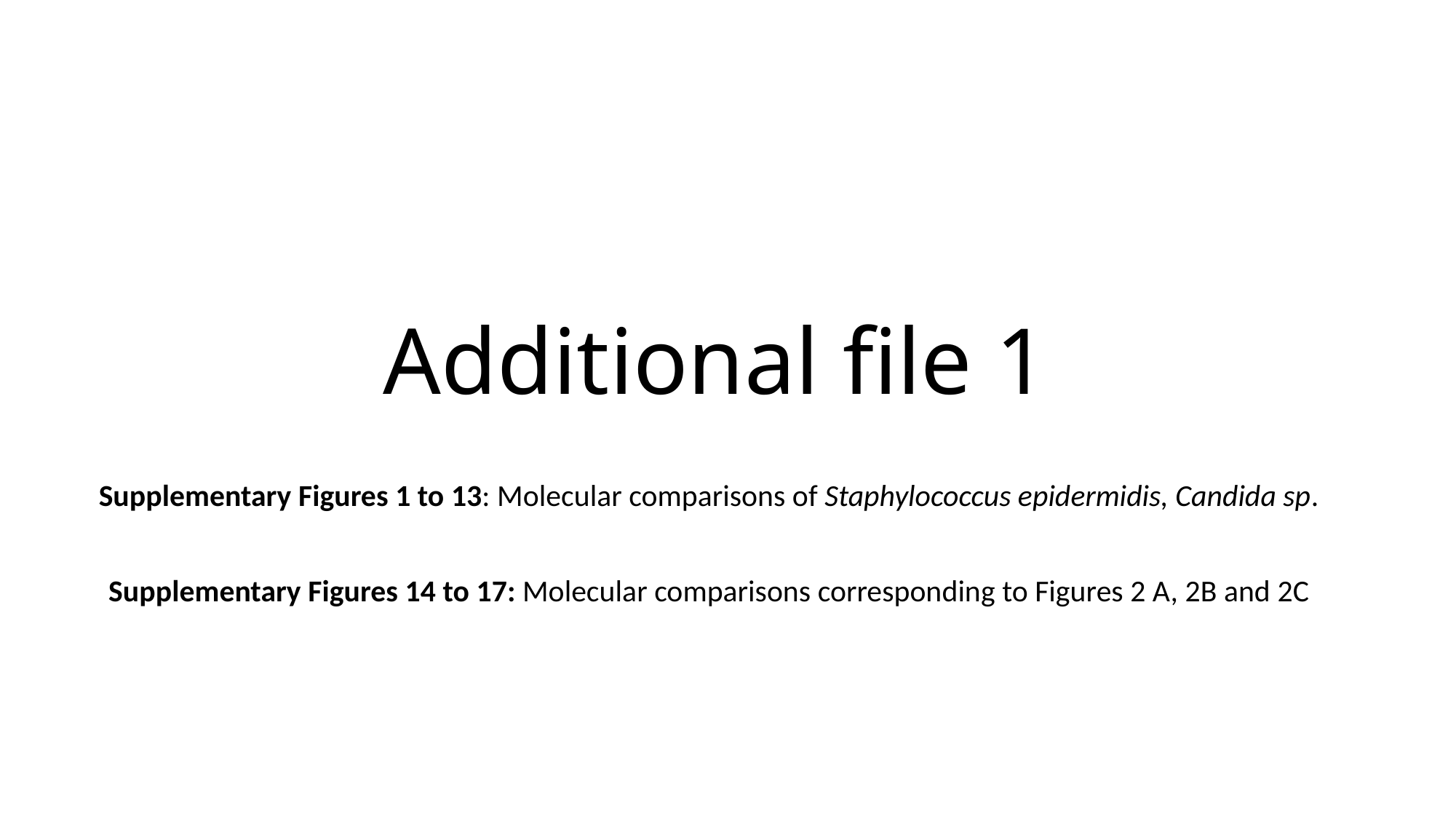

# Additional file 1
Supplementary Figures 1 to 13: Molecular comparisons of Staphylococcus epidermidis, Candida sp.
Supplementary Figures 14 to 17: Molecular comparisons corresponding to Figures 2 A, 2B and 2C

## Slide 3
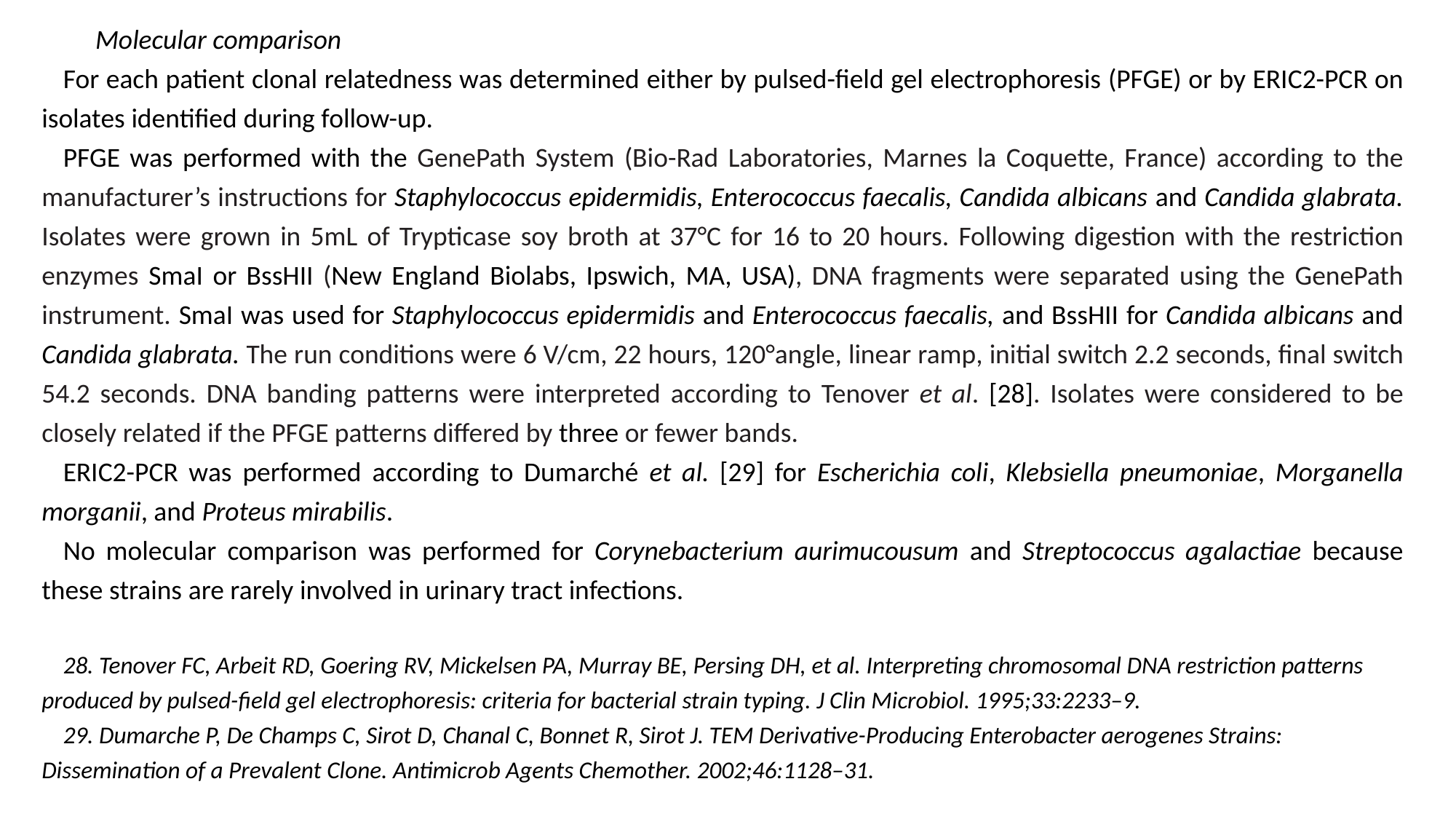

Molecular comparison
For each patient clonal relatedness was determined either by pulsed-field gel electrophoresis (PFGE) or by ERIC2-PCR on isolates identified during follow-up.
PFGE was performed with the GenePath System (Bio-Rad Laboratories, Marnes la Coquette, France) according to the manufacturer’s instructions for Staphylococcus epidermidis, Enterococcus faecalis, Candida albicans and Candida glabrata. Isolates were grown in 5mL of Trypticase soy broth at 37°C for 16 to 20 hours. Following digestion with the restriction enzymes SmaI or BssHII (New England Biolabs, Ipswich, MA, USA), DNA fragments were separated using the GenePath instrument. SmaI was used for Staphylococcus epidermidis and Enterococcus faecalis, and BssHII for Candida albicans and Candida glabrata. The run conditions were 6 V/cm, 22 hours, 120°angle, linear ramp, initial switch 2.2 seconds, final switch 54.2 seconds. DNA banding patterns were interpreted according to Tenover et al. [28]. Isolates were considered to be closely related if the PFGE patterns differed by three or fewer bands.
ERIC2-PCR was performed according to Dumarché et al. [29] for Escherichia coli, Klebsiella pneumoniae, Morganella morganii, and Proteus mirabilis.
No molecular comparison was performed for Corynebacterium aurimucousum and Streptococcus agalactiae because these strains are rarely involved in urinary tract infections.
28. Tenover FC, Arbeit RD, Goering RV, Mickelsen PA, Murray BE, Persing DH, et al. Interpreting chromosomal DNA restriction patterns produced by pulsed-field gel electrophoresis: criteria for bacterial strain typing. J Clin Microbiol. 1995;33:2233–9.
29. Dumarche P, De Champs C, Sirot D, Chanal C, Bonnet R, Sirot J. TEM Derivative-Producing Enterobacter aerogenes Strains: Dissemination of a Prevalent Clone. Antimicrob Agents Chemother. 2002;46:1128–31.

## Slide 4
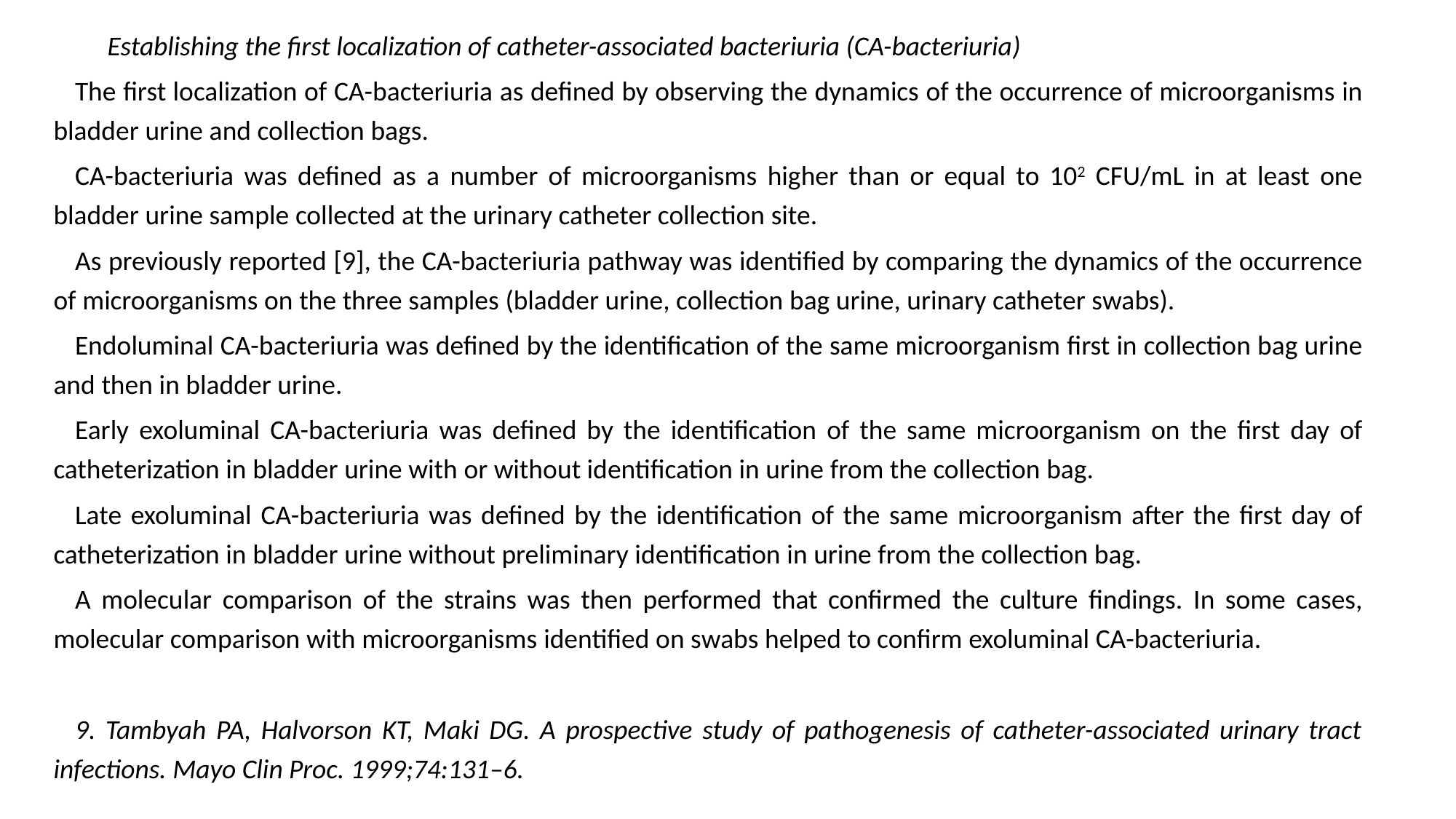

Establishing the first localization of catheter-associated bacteriuria (CA-bacteriuria)
The first localization of CA-bacteriuria as defined by observing the dynamics of the occurrence of microorganisms in bladder urine and collection bags.
CA-bacteriuria was defined as a number of microorganisms higher than or equal to 102 CFU/mL in at least one bladder urine sample collected at the urinary catheter collection site.
As previously reported [9], the CA-bacteriuria pathway was identified by comparing the dynamics of the occurrence of microorganisms on the three samples (bladder urine, collection bag urine, urinary catheter swabs).
Endoluminal CA-bacteriuria was defined by the identification of the same microorganism first in collection bag urine and then in bladder urine.
Early exoluminal CA-bacteriuria was defined by the identification of the same microorganism on the first day of catheterization in bladder urine with or without identification in urine from the collection bag.
Late exoluminal CA-bacteriuria was defined by the identification of the same microorganism after the first day of catheterization in bladder urine without preliminary identification in urine from the collection bag.
A molecular comparison of the strains was then performed that confirmed the culture findings. In some cases, molecular comparison with microorganisms identified on swabs helped to confirm exoluminal CA-bacteriuria.
9. Tambyah PA, Halvorson KT, Maki DG. A prospective study of pathogenesis of catheter-associated urinary tract infections. Mayo Clin Proc. 1999;74:131–6.

## Slide 5
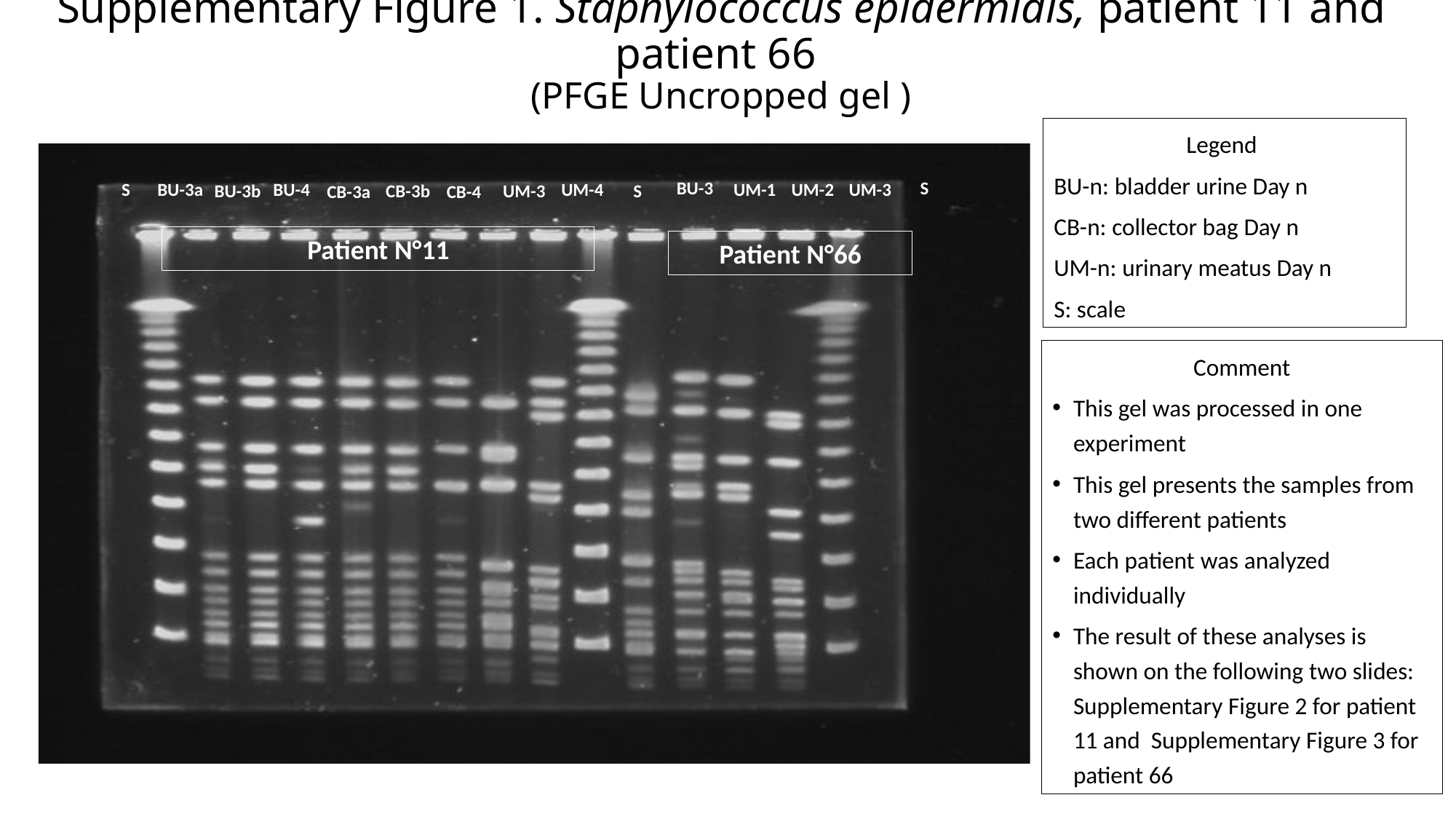

# Supplementary Figure 1. Staphylococcus epidermidis, patient 11 and patient 66 (PFGE Uncropped gel )
Legend
BU-n: bladder urine Day n
CB-n: collector bag Day n
UM-n: urinary meatus Day n
S: scale
BU-3
S
S
BU-4
UM-1
UM-2
UM-3
UM-4
BU-3a
BU-3b
CB-3b
S
UM-3
CB-3a
CB-4
Patient N°11
Patient N°66
Comment
This gel was processed in one experiment
This gel presents the samples from two different patients
Each patient was analyzed individually
The result of these analyses is shown on the following two slides: Supplementary Figure 2 for patient 11 and Supplementary Figure 3 for patient 66

## Slide 6
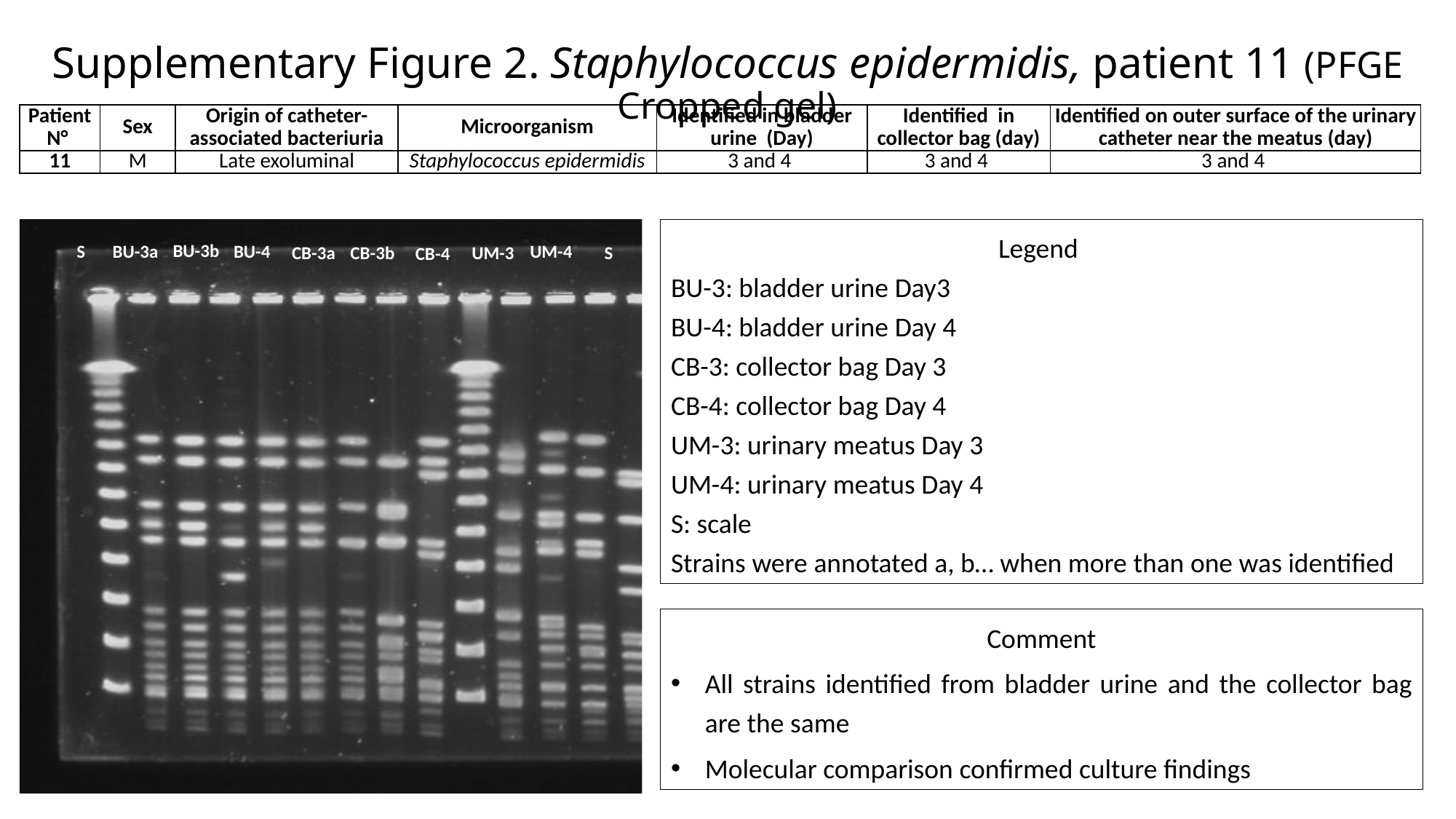

Supplementary Figure 2. Staphylococcus epidermidis, patient 11 (PFGE Cropped gel)
| Patient N° | Sex | Origin of catheter-associated bacteriuria | Microorganism | Identified in bladder urine (Day) | Identified in collector bag (day) | Identified on outer surface of the urinary catheter near the meatus (day) |
| --- | --- | --- | --- | --- | --- | --- |
| 11 | M | Late exoluminal | Staphylococcus epidermidis | 3 and 4 | 3 and 4 | 3 and 4 |
BU-3b
BU-4
UM-4
S
BU-3a
CB-3b
S
CB-3a
UM-3
CB-4
Legend
BU-3: bladder urine Day3
BU-4: bladder urine Day 4
CB-3: collector bag Day 3
CB-4: collector bag Day 4
UM-3: urinary meatus Day 3
UM-4: urinary meatus Day 4
S: scale
Strains were annotated a, b… when more than one was identified
Comment
All strains identified from bladder urine and the collector bag are the same
Molecular comparison confirmed culture findings

## Slide 7
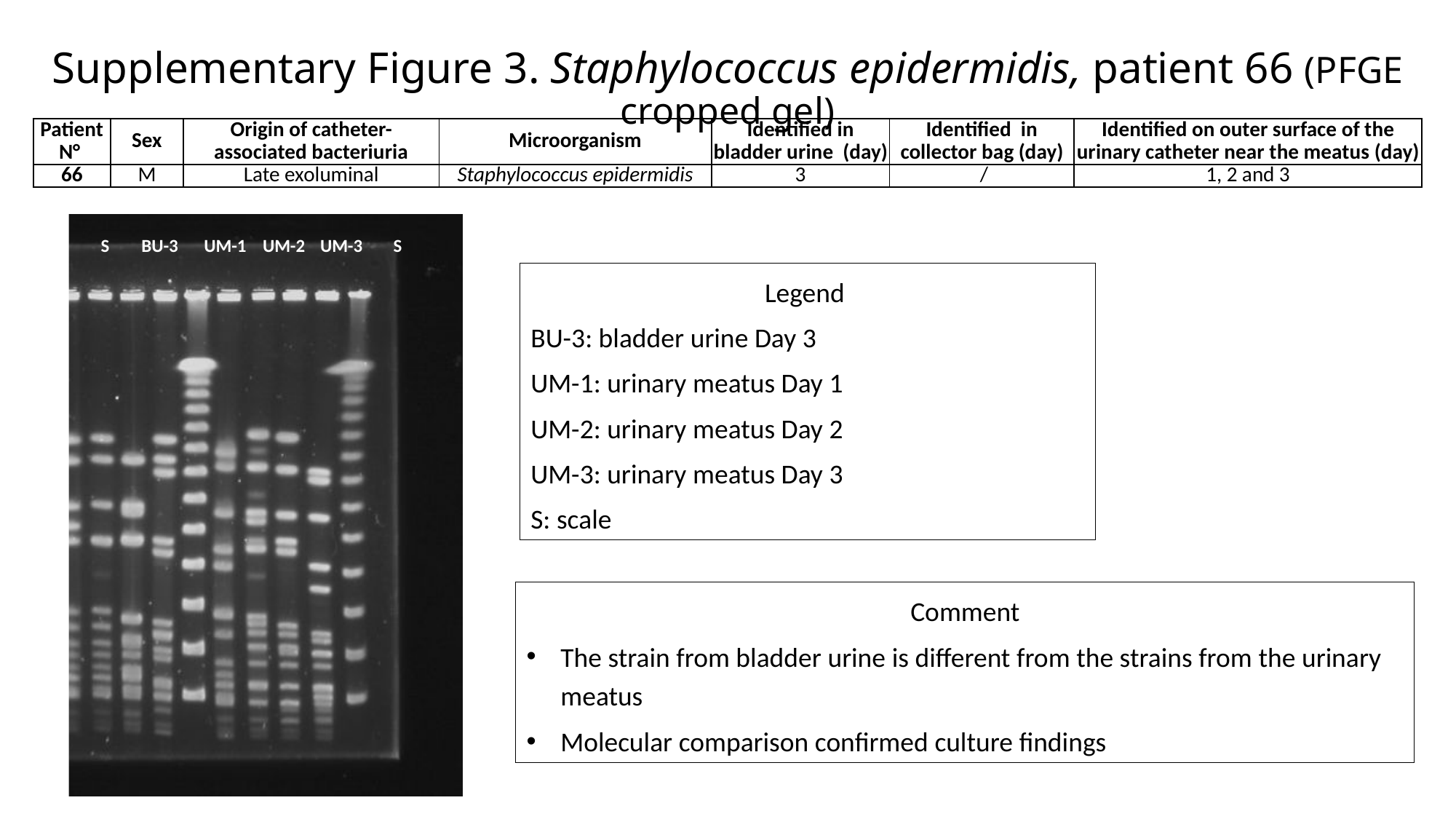

Supplementary Figure 3. Staphylococcus epidermidis, patient 66 (PFGE cropped gel)
| Patient N° | Sex | Origin of catheter-associated bacteriuria | Microorganism | Identified in bladder urine (day) | Identified in collector bag (day) | Identified on outer surface of the urinary catheter near the meatus (day) |
| --- | --- | --- | --- | --- | --- | --- |
| 66 | M | Late exoluminal | Staphylococcus epidermidis | 3 | / | 1, 2 and 3 |
UM-3
UM-2
UM-1
BU-3
S
S
Legend
BU-3: bladder urine Day 3
UM-1: urinary meatus Day 1
UM-2: urinary meatus Day 2
UM-3: urinary meatus Day 3
S: scale
Comment
The strain from bladder urine is different from the strains from the urinary meatus
Molecular comparison confirmed culture findings

## Slide 8
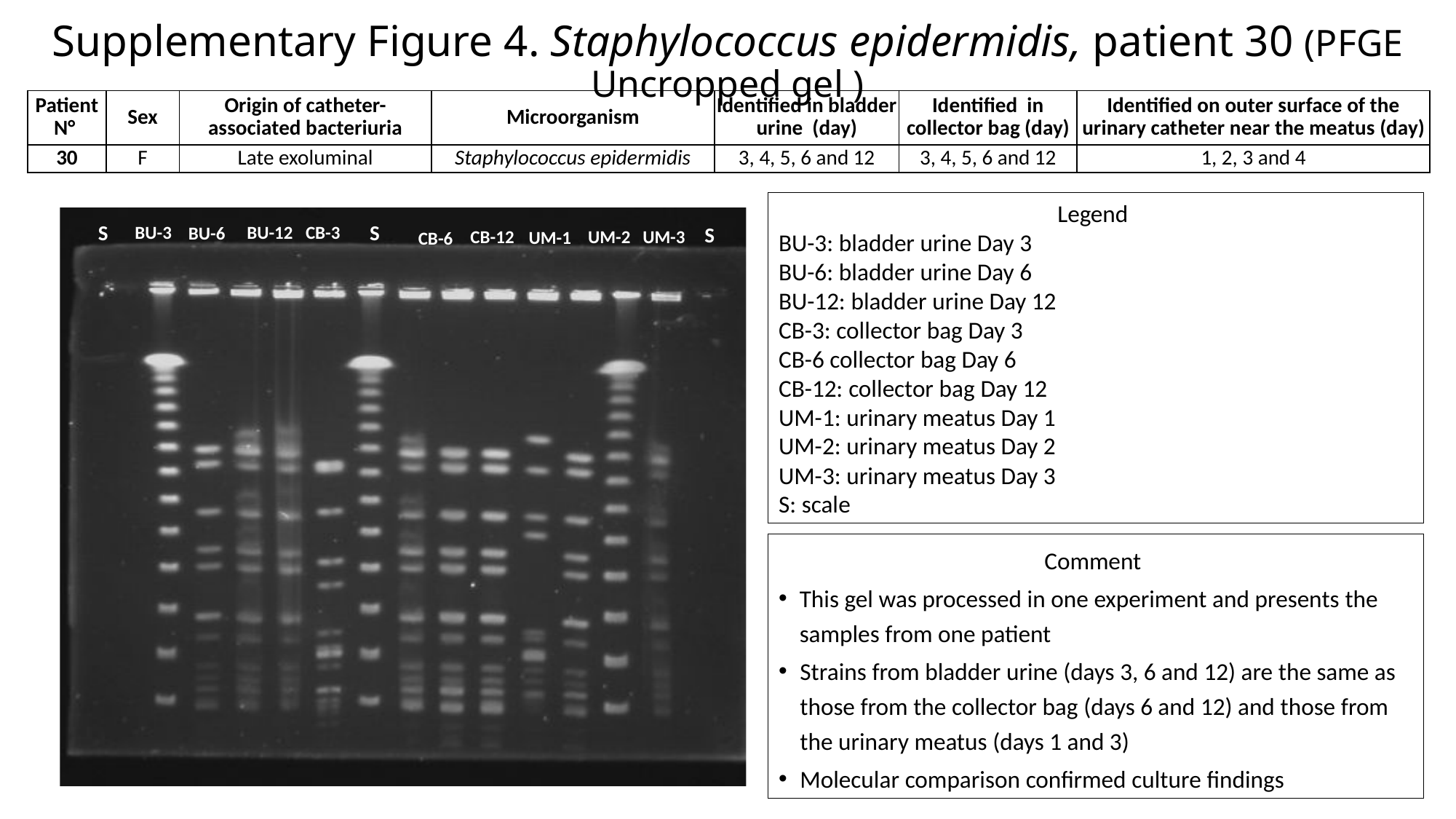

Supplementary Figure 4. Staphylococcus epidermidis, patient 30 (PFGE Uncropped gel )
| Patient N° | Sex | Origin of catheter-associated bacteriuria | Microorganism | Identified in bladder urine (day) | Identified in collector bag (day) | Identified on outer surface of the urinary catheter near the meatus (day) |
| --- | --- | --- | --- | --- | --- | --- |
| 30 | F | Late exoluminal | Staphylococcus epidermidis | 3, 4, 5, 6 and 12 | 3, 4, 5, 6 and 12 | 1, 2, 3 and 4 |
Legend
BU-3: bladder urine Day 3
BU-6: bladder urine Day 6
BU-12: bladder urine Day 12
CB-3: collector bag Day 3
CB-6 collector bag Day 6
CB-12: collector bag Day 12
UM-1: urinary meatus Day 1
UM-2: urinary meatus Day 2
UM-3: urinary meatus Day 3
S: scale
S
S
CB-3
BU-12
BU-3
BU-6
S
CB-12
UM-2
UM-3
UM-1
CB-6
Comment
This gel was processed in one experiment and presents the samples from one patient
Strains from bladder urine (days 3, 6 and 12) are the same as those from the collector bag (days 6 and 12) and those from the urinary meatus (days 1 and 3)
Molecular comparison confirmed culture findings

## Slide 9
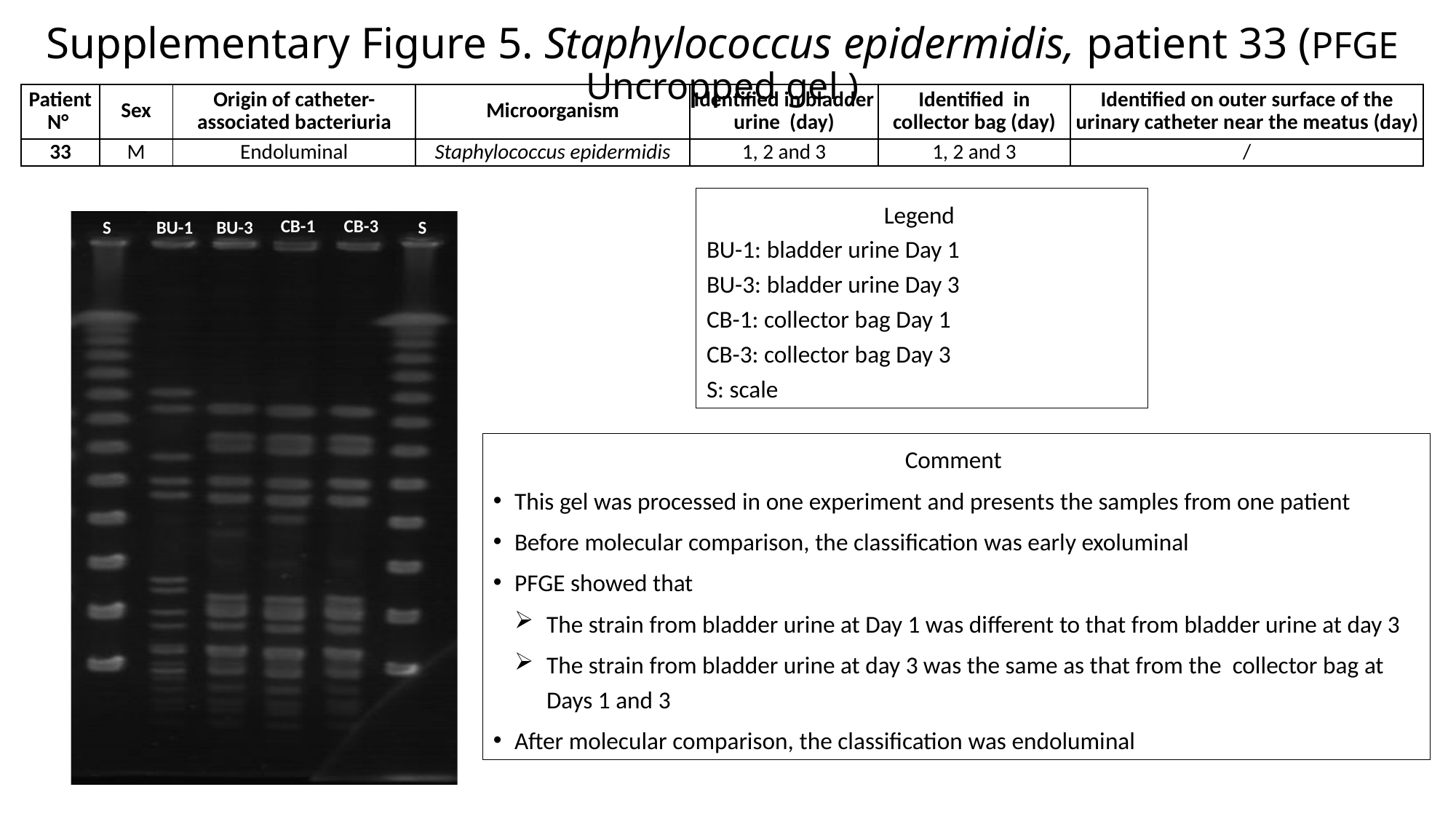

Supplementary Figure 5. Staphylococcus epidermidis, patient 33 (PFGE Uncropped gel )
| Patient N° | Sex | Origin of catheter-associated bacteriuria | Microorganism | Identified in bladder urine (day) | Identified in collector bag (day) | Identified on outer surface of the urinary catheter near the meatus (day) |
| --- | --- | --- | --- | --- | --- | --- |
| 33 | M | Endoluminal | Staphylococcus epidermidis | 1, 2 and 3 | 1, 2 and 3 | / |
Legend
BU-1: bladder urine Day 1
BU-3: bladder urine Day 3
CB-1: collector bag Day 1
CB-3: collector bag Day 3
S: scale
CB-1
CB-3
BU-3
BU-1
S
S
Comment
This gel was processed in one experiment and presents the samples from one patient
Before molecular comparison, the classification was early exoluminal
PFGE showed that
The strain from bladder urine at Day 1 was different to that from bladder urine at day 3
The strain from bladder urine at day 3 was the same as that from the collector bag at Days 1 and 3
After molecular comparison, the classification was endoluminal

## Slide 10
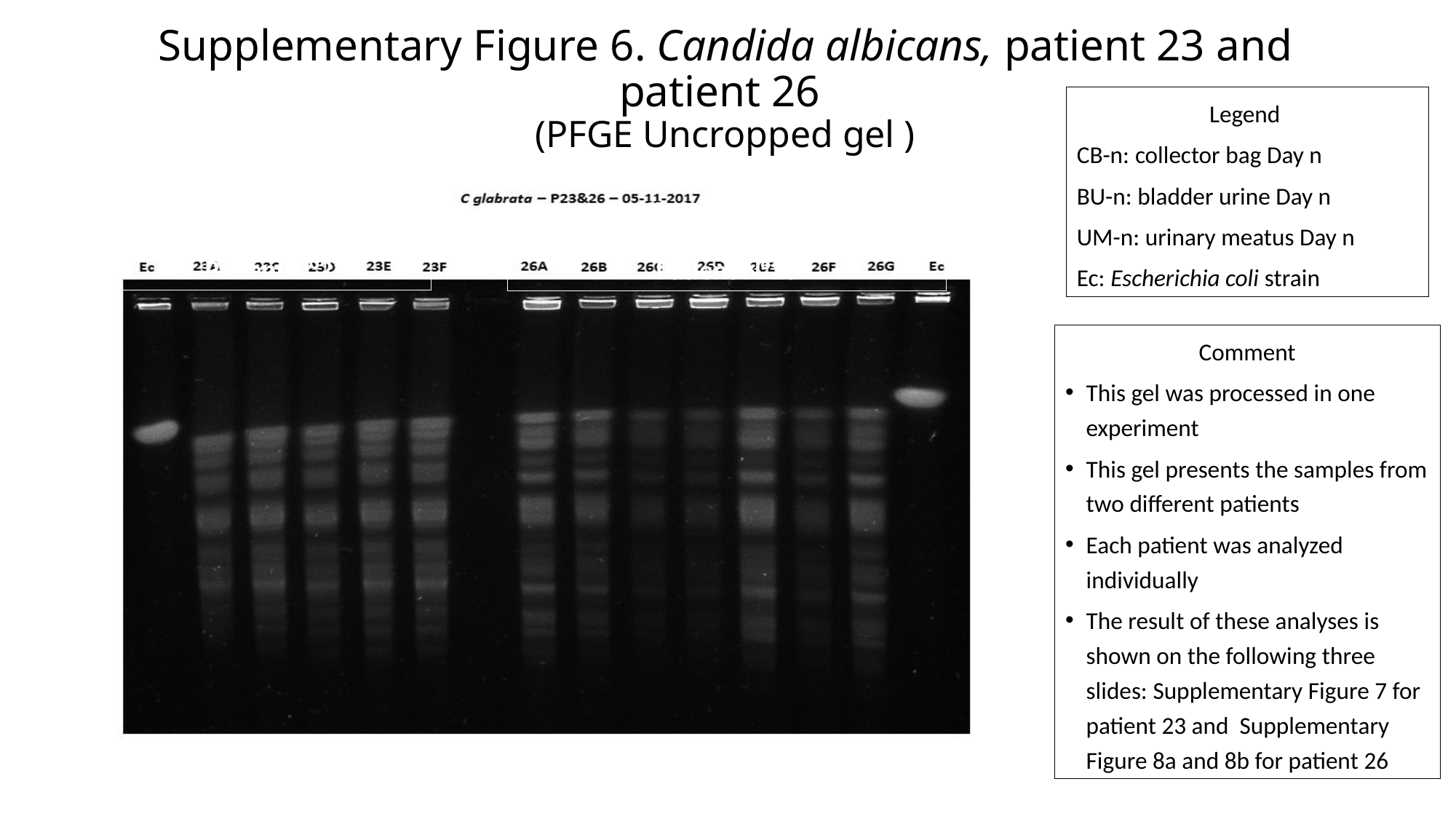

Supplementary Figure 6. Candida albicans, patient 23 and patient 26
(PFGE Uncropped gel )
Legend
CB-n: collector bag Day n
BU-n: bladder urine Day n
UM-n: urinary meatus Day n
Ec: Escherichia coli strain
Ec
CB-3
CB-1
BU-3
CB-12
BU-5
Ec
BU-6
BU-9
BU-12
BU-1
BU-2
CB-9
CB-6
Patient N°23
Patient N°26
Comment
This gel was processed in one experiment
This gel presents the samples from two different patients
Each patient was analyzed individually
The result of these analyses is shown on the following three slides: Supplementary Figure 7 for patient 23 and Supplementary Figure 8a and 8b for patient 26

## Slide 11
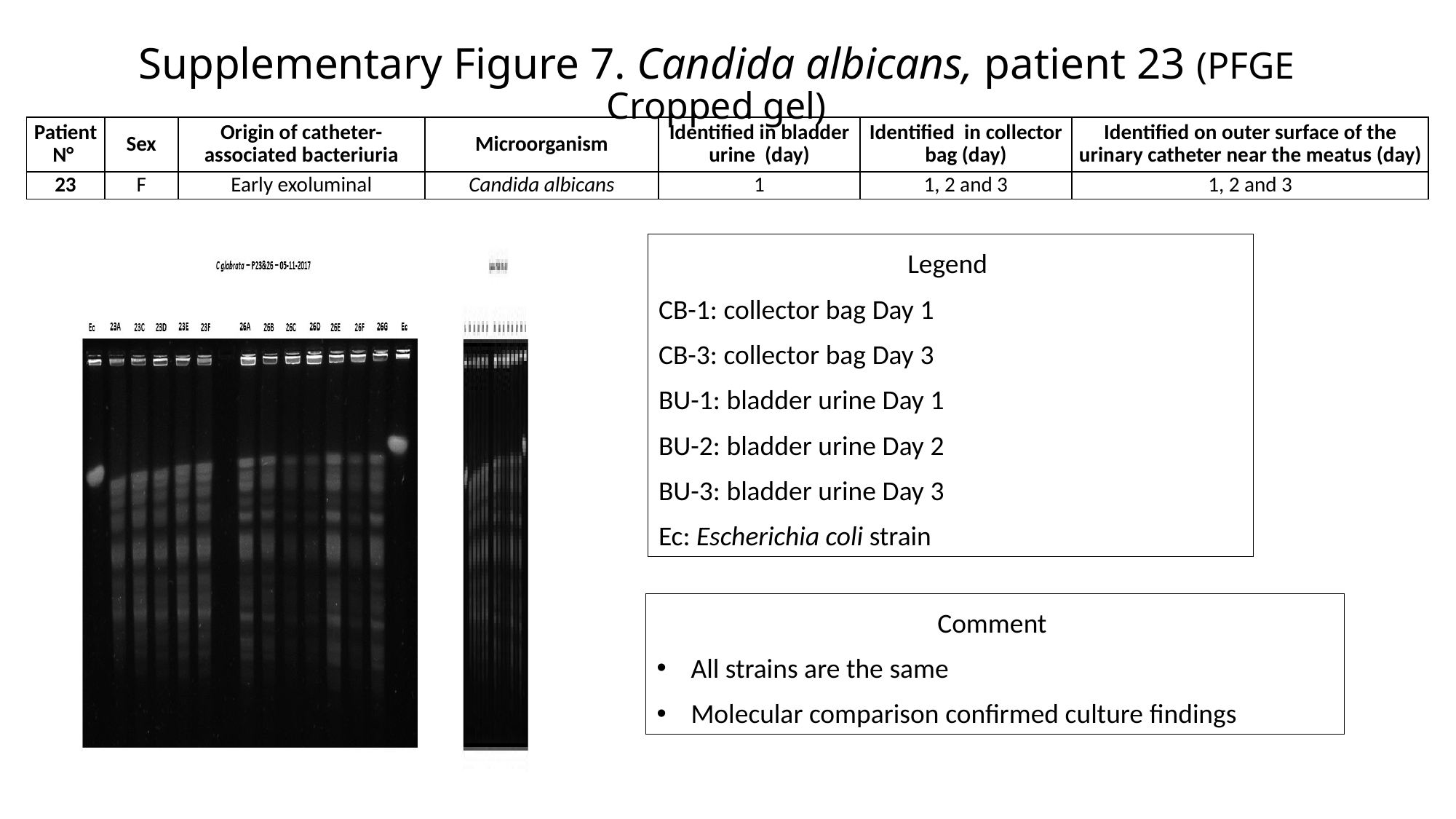

Supplementary Figure 7. Candida albicans, patient 23 (PFGE Cropped gel)
| Patient N° | Sex | Origin of catheter-associated bacteriuria | Microorganism | Identified in bladder urine (day) | Identified in collector bag (day) | Identified on outer surface of the urinary catheter near the meatus (day) |
| --- | --- | --- | --- | --- | --- | --- |
| 23 | F | Early exoluminal | Candida albicans | 1 | 1, 2 and 3 | 1, 2 and 3 |
Ec
BU-3
CB-3
Ec
CB-1
BU-2
BU-1
Legend
CB-1: collector bag Day 1
CB-3: collector bag Day 3
BU-1: bladder urine Day 1
BU-2: bladder urine Day 2
BU-3: bladder urine Day 3
Ec: Escherichia coli strain
Comment
All strains are the same
Molecular comparison confirmed culture findings

## Slide 12
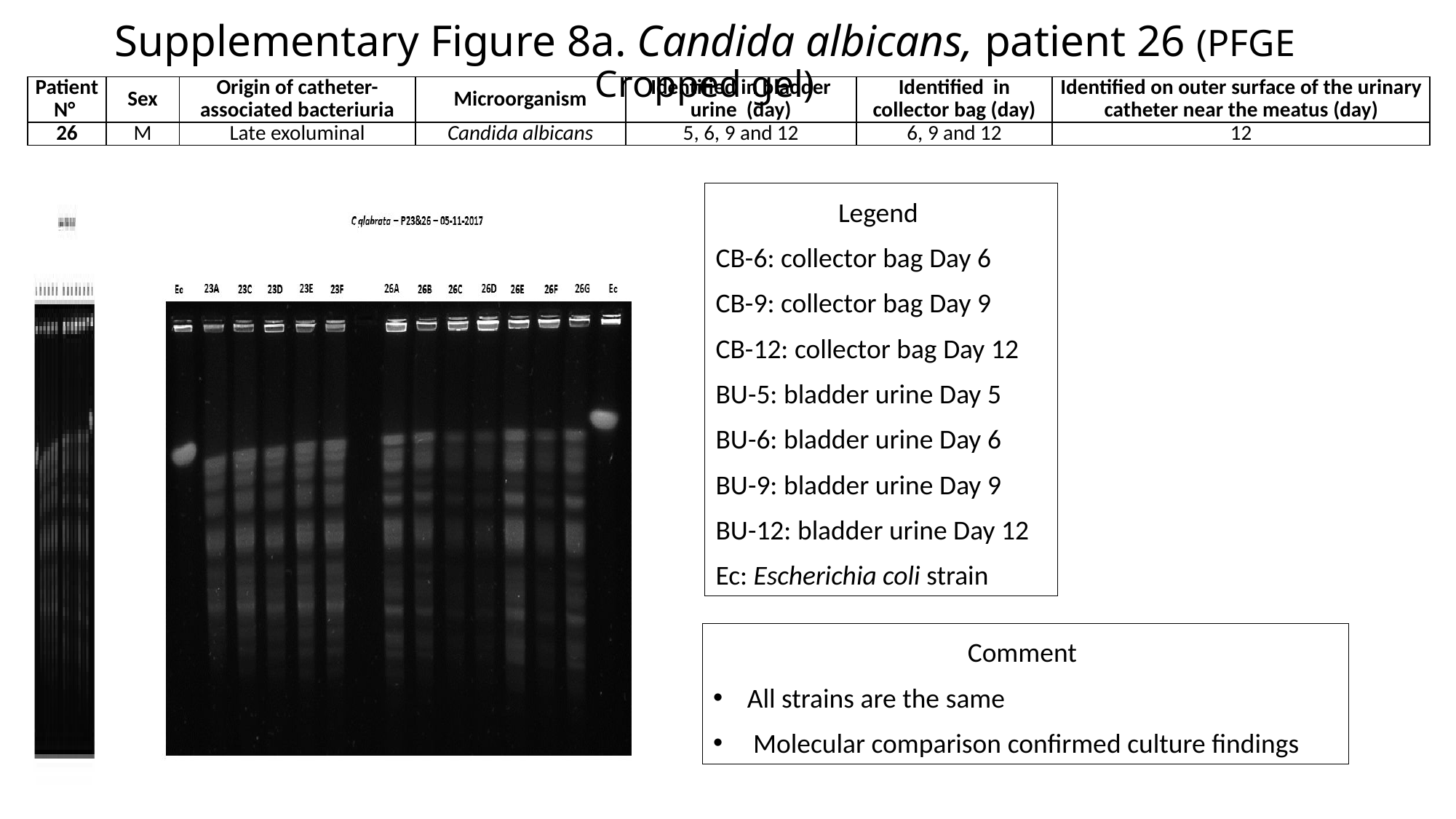

Supplementary Figure 8a. Candida albicans, patient 26 (PFGE Cropped gel)
| Patient N° | Sex | Origin of catheter-associated bacteriuria | Microorganism | Identified in bladder urine (day) | Identified in collector bag (day) | Identified on outer surface of the urinary catheter near the meatus (day) |
| --- | --- | --- | --- | --- | --- | --- |
| 26 | M | Late exoluminal | Candida albicans | 5, 6, 9 and 12 | 6, 9 and 12 | 12 |
Ec
Ec
CB-12
BU-5
BU-6
BU-9
BU-12
CB-9
CB-6
Legend
CB-6: collector bag Day 6
CB-9: collector bag Day 9
CB-12: collector bag Day 12
BU-5: bladder urine Day 5
BU-6: bladder urine Day 6
BU-9: bladder urine Day 9
BU-12: bladder urine Day 12
Ec: Escherichia coli strain
Comment
All strains are the same
 Molecular comparison confirmed culture findings

## Slide 13
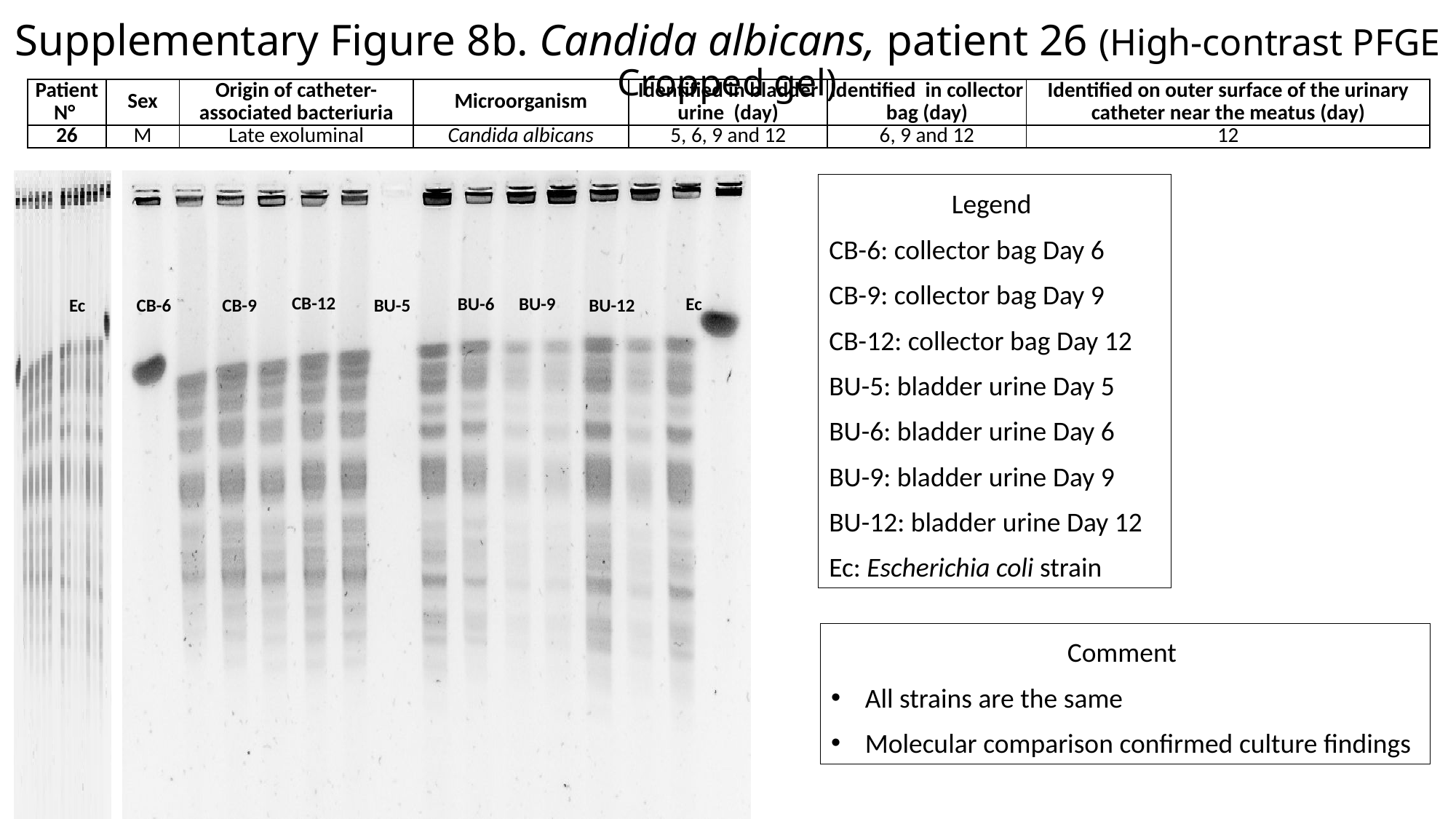

Supplementary Figure 8b. Candida albicans, patient 26 (High-contrast PFGE Cropped gel)
| Patient N° | Sex | Origin of catheter-associated bacteriuria | Microorganism | Identified in bladder urine (day) | Identified in collector bag (day) | Identified on outer surface of the urinary catheter near the meatus (day) |
| --- | --- | --- | --- | --- | --- | --- |
| 26 | M | Late exoluminal | Candida albicans | 5, 6, 9 and 12 | 6, 9 and 12 | 12 |
CB-12
BU-6
BU-9
Ec
Ec
BU-12
BU-5
CB-6
CB-9
Legend
CB-6: collector bag Day 6
CB-9: collector bag Day 9
CB-12: collector bag Day 12
BU-5: bladder urine Day 5
BU-6: bladder urine Day 6
BU-9: bladder urine Day 9
BU-12: bladder urine Day 12
Ec: Escherichia coli strain
Comment
All strains are the same
Molecular comparison confirmed culture findings

## Slide 14
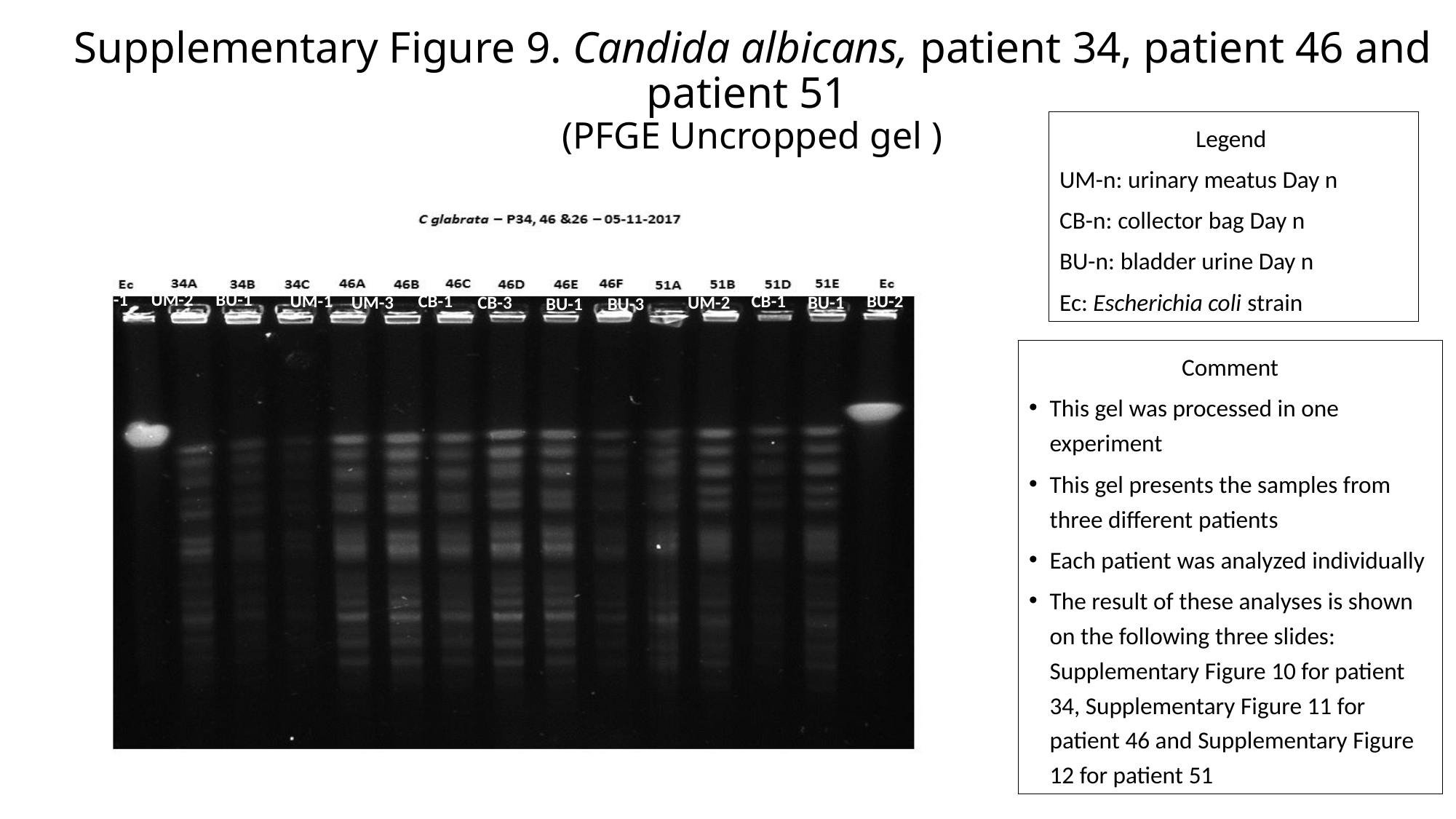

Supplementary Figure 9. Candida albicans, patient 34, patient 46 and patient 51
(PFGE Uncropped gel )
Legend
UM-n: urinary meatus Day n
CB-n: collector bag Day n
BU-n: bladder urine Day n
Ec: Escherichia coli strain
Patient N°51
Patient N°46
Patient N°34
Ec
Ec
UM-1
UM-2
BU-1
UM-1
CB-1
CB-1
BU-2
UM-3
CB-3
UM-2
BU-1
BU-3
BU-1
Comment
This gel was processed in one experiment
This gel presents the samples from three different patients
Each patient was analyzed individually
The result of these analyses is shown on the following three slides: Supplementary Figure 10 for patient 34, Supplementary Figure 11 for patient 46 and Supplementary Figure 12 for patient 51

## Slide 15
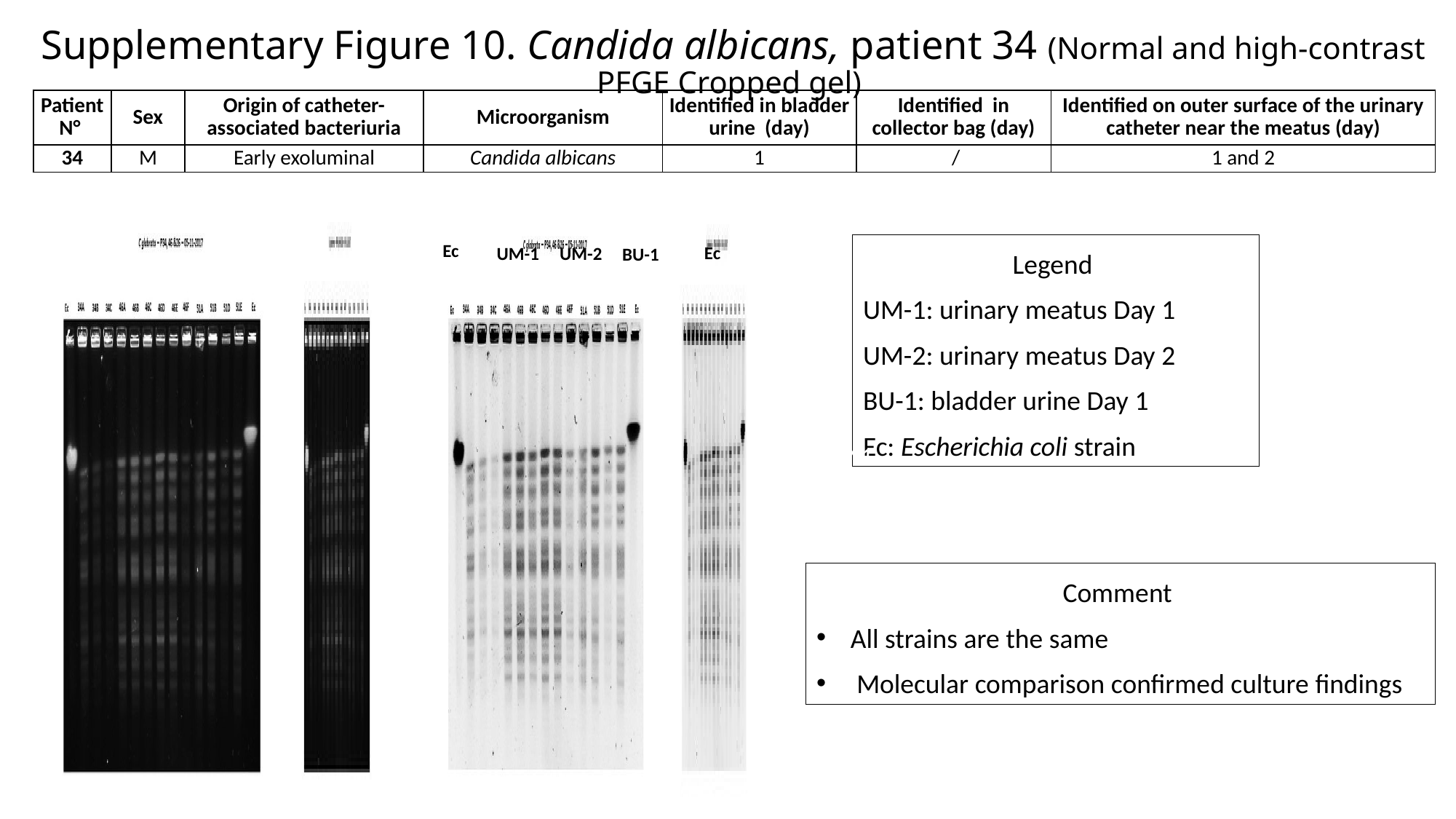

Supplementary Figure 10. Candida albicans, patient 34 (Normal and high-contrast PFGE Cropped gel)
| Patient N° | Sex | Origin of catheter-associated bacteriuria | Microorganism | Identified in bladder urine (day) | Identified in collector bag (day) | Identified on outer surface of the urinary catheter near the meatus (day) |
| --- | --- | --- | --- | --- | --- | --- |
| 34 | M | Early exoluminal | Candida albicans | 1 | / | 1 and 2 |
Ec
Ec
UM-2
BU-1
UM-1
Ec
Ec
UM-2
UM-1
BU-1
Legend
UM-1: urinary meatus Day 1
UM-2: urinary meatus Day 2
BU-1: bladder urine Day 1
Ec: Escherichia coli strain
UM-1
Comment
All strains are the same
 Molecular comparison confirmed culture findings

## Slide 16
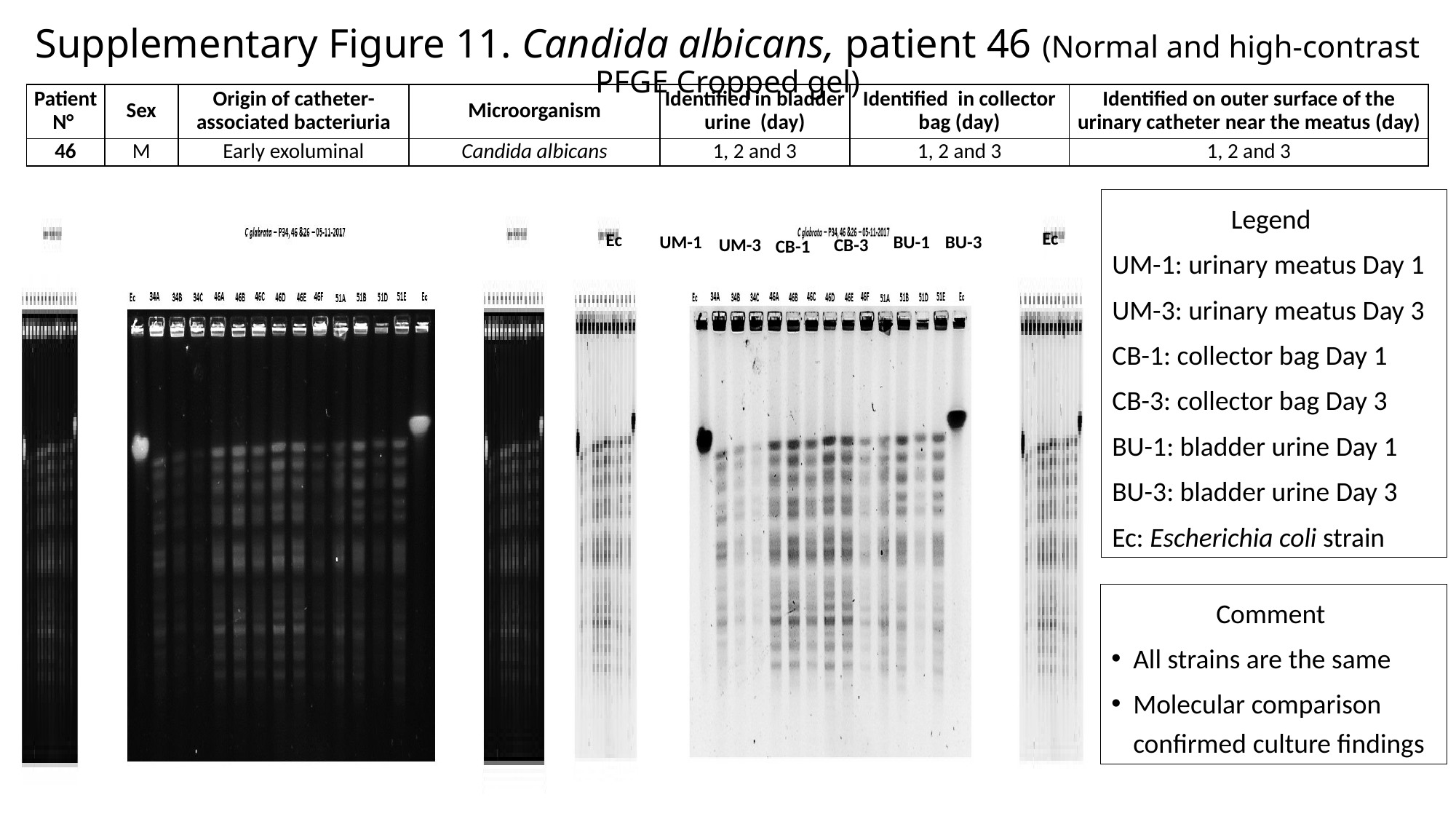

Supplementary Figure 11. Candida albicans, patient 46 (Normal and high-contrast PFGE Cropped gel)
| Patient N° | Sex | Origin of catheter-associated bacteriuria | Microorganism | Identified in bladder urine (day) | Identified in collector bag (day) | Identified on outer surface of the urinary catheter near the meatus (day) |
| --- | --- | --- | --- | --- | --- | --- |
| 46 | M | Early exoluminal | Candida albicans | 1, 2 and 3 | 1, 2 and 3 | 1, 2 and 3 |
Ec
Ec
UM-1
BU-1
BU-3
CB-3
UM-3
CB-1
Ec
Ec
BU-3
UM-1
BU-1
CB-3
UM-3
CB-1
Legend
UM-1: urinary meatus Day 1
UM-3: urinary meatus Day 3
CB-1: collector bag Day 1
CB-3: collector bag Day 3
BU-1: bladder urine Day 1
BU-3: bladder urine Day 3
Ec: Escherichia coli strain
Comment
All strains are the same
Molecular comparison confirmed culture findings

## Slide 17
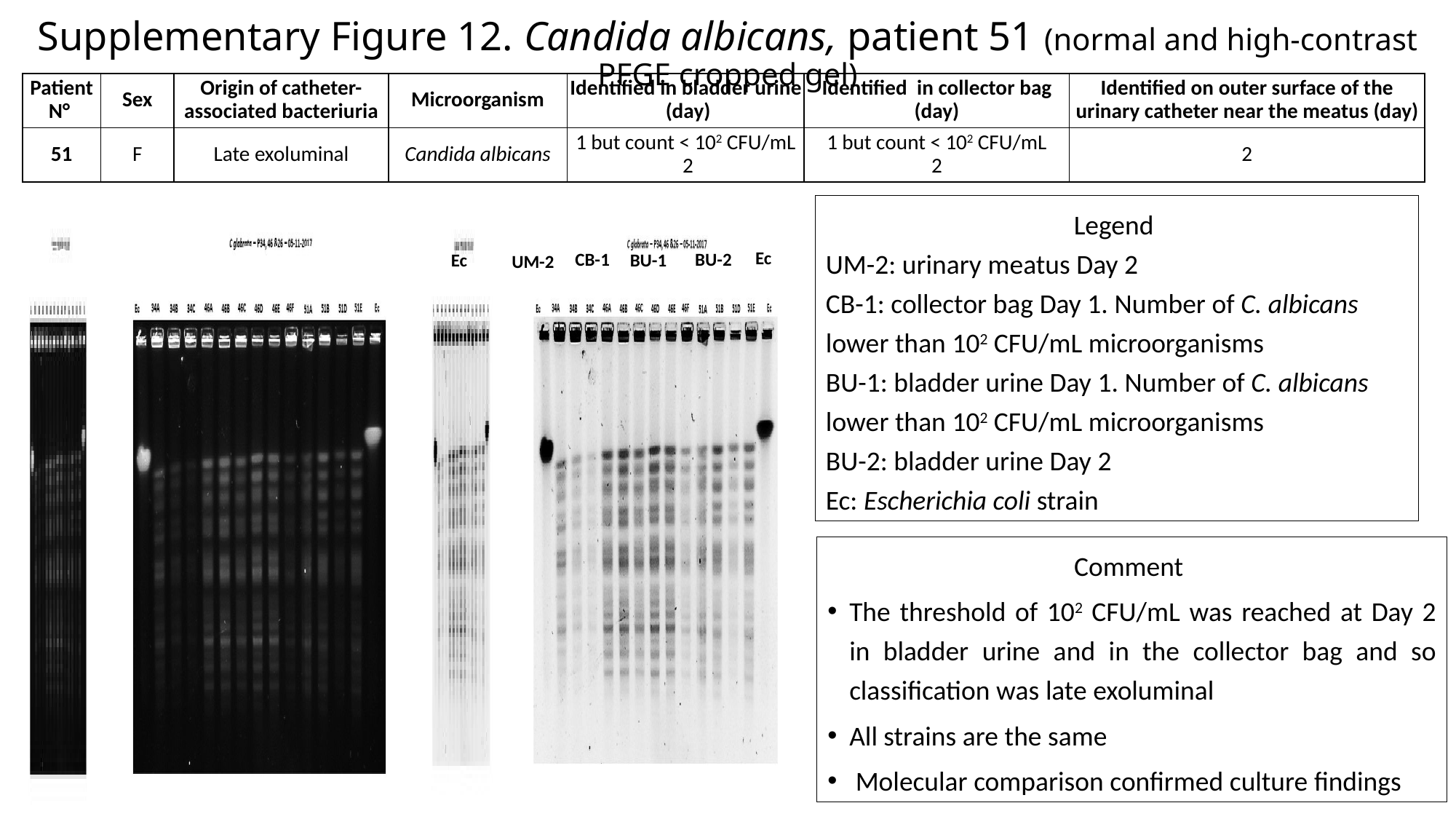

Supplementary Figure 12. Candida albicans, patient 51 (normal and high-contrast PFGE cropped gel)
| Patient N° | Sex | Origin of catheter-associated bacteriuria | Microorganism | Identified in bladder urine (day) | Identified in collector bag (day) | Identified on outer surface of the urinary catheter near the meatus (day) |
| --- | --- | --- | --- | --- | --- | --- |
| 51 | F | Late exoluminal | Candida albicans | 1 but count < 102 CFU/mL 2 | 1 but count < 102 CFU/mL 2 | 2 |
Ec
Ec
UM-2
BU-1
CB-1
BU-2
Legend
UM-2: urinary meatus Day 2
CB-1: collector bag Day 1. Number of C. albicans lower than 102 CFU/mL microorganisms
BU-1: bladder urine Day 1. Number of C. albicans lower than 102 CFU/mL microorganisms
BU-2: bladder urine Day 2
Ec: Escherichia coli strain
Ec
BU-2
CB-1
Ec
BU-1
UM-2
Comment
The threshold of 102 CFU/mL was reached at Day 2 in bladder urine and in the collector bag and so classification was late exoluminal
All strains are the same
 Molecular comparison confirmed culture findings

## Slide 18
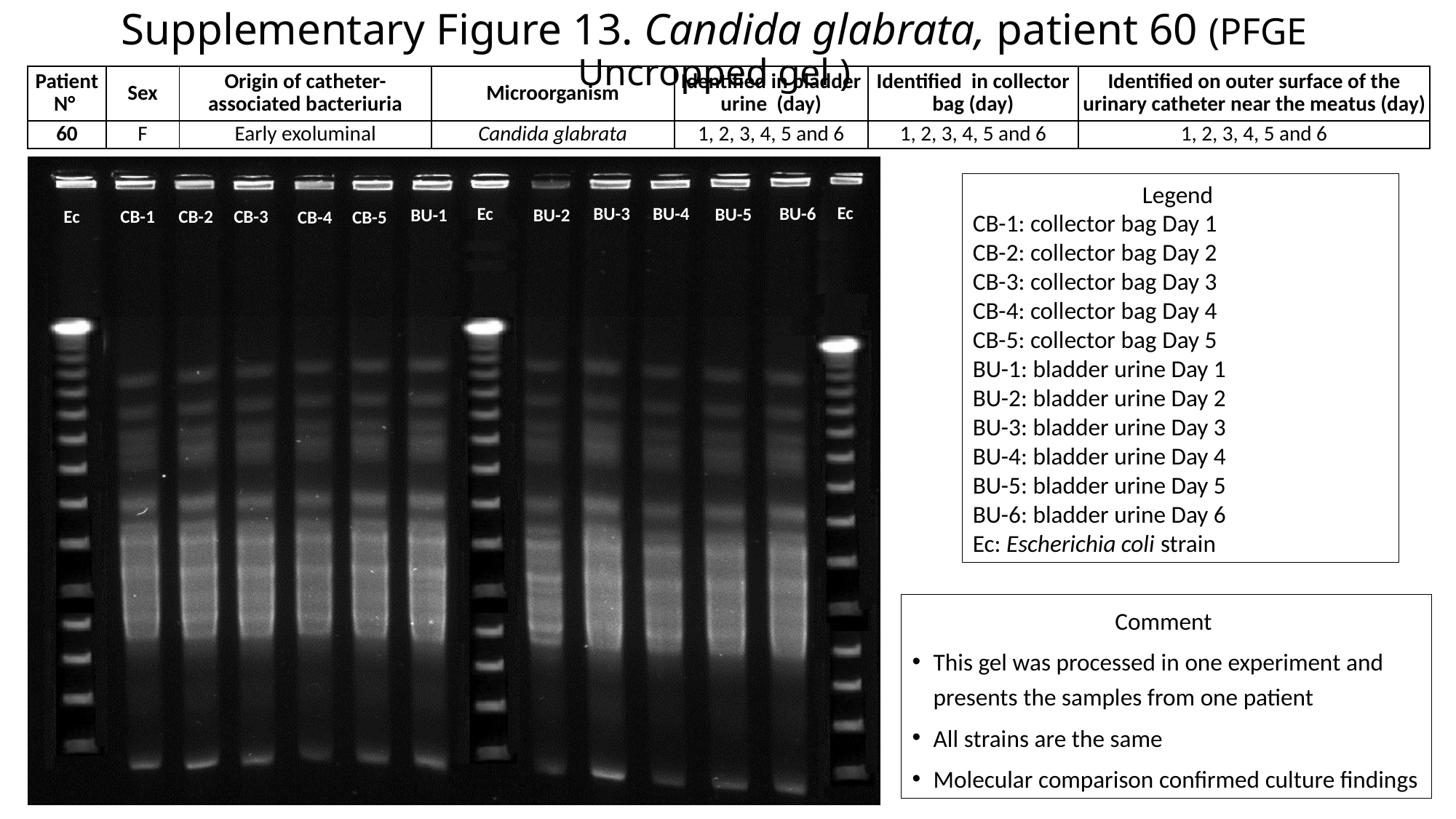

Supplementary Figure 13. Candida glabrata, patient 60 (PFGE Uncropped gel )
| Patient N° | Sex | Origin of catheter-associated bacteriuria | Microorganism | Identified in bladder urine (day) | Identified in collector bag (day) | Identified on outer surface of the urinary catheter near the meatus (day) |
| --- | --- | --- | --- | --- | --- | --- |
| 60 | F | Early exoluminal | Candida glabrata | 1, 2, 3, 4, 5 and 6 | 1, 2, 3, 4, 5 and 6 | 1, 2, 3, 4, 5 and 6 |
Ec
BU-6
Ec
BU-4
BU-3
BU-5
BU-2
BU-1
CB-2
CB-3
Ec
CB-1
CB-5
CB-4
Legend
CB-1: collector bag Day 1
CB-2: collector bag Day 2
CB-3: collector bag Day 3
CB-4: collector bag Day 4
CB-5: collector bag Day 5
BU-1: bladder urine Day 1
BU-2: bladder urine Day 2
BU-3: bladder urine Day 3
BU-4: bladder urine Day 4
BU-5: bladder urine Day 5
BU-6: bladder urine Day 6
Ec: Escherichia coli strain
Comment
This gel was processed in one experiment and presents the samples from one patient
All strains are the same
Molecular comparison confirmed culture findings

## Slide 19
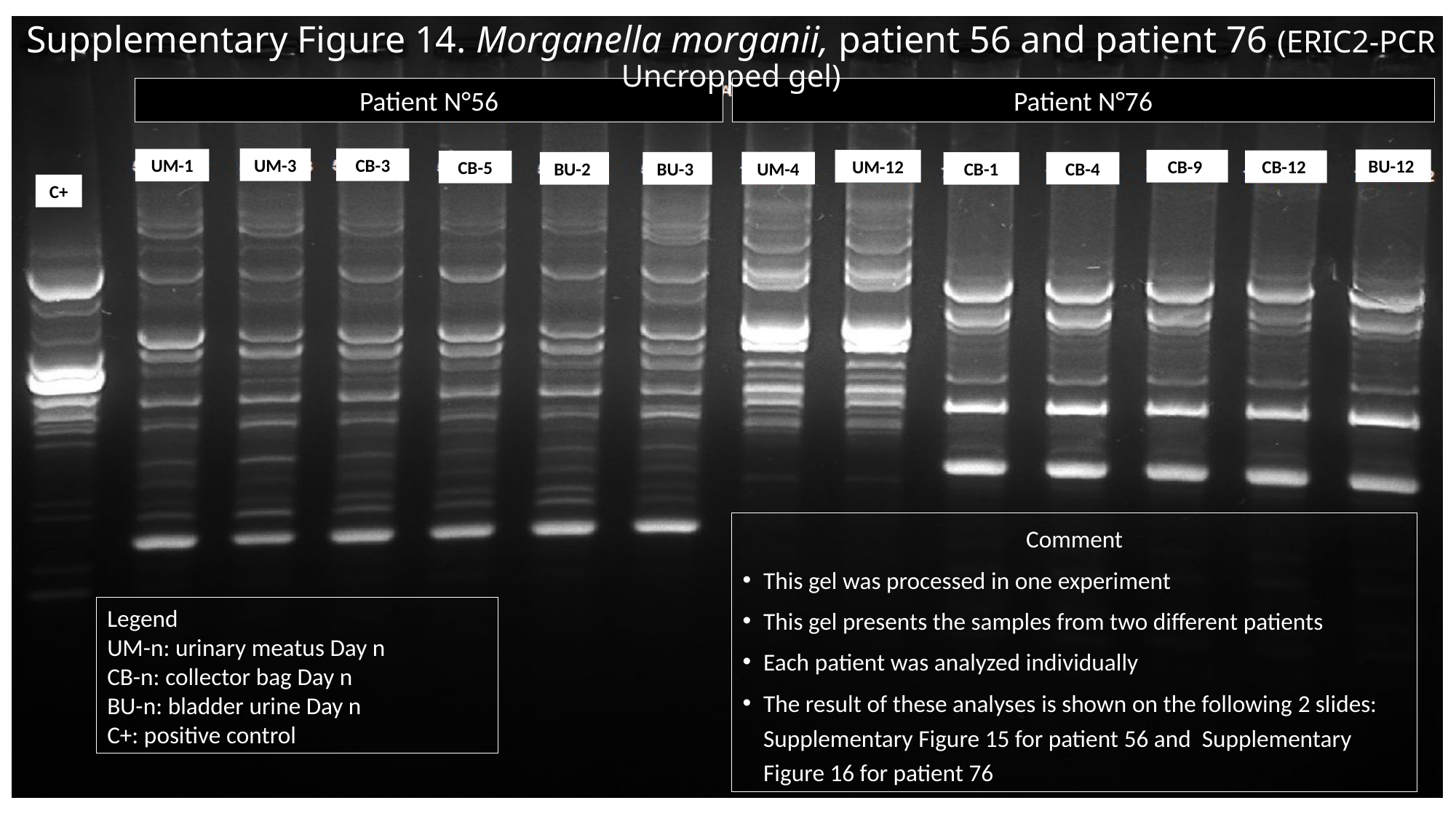

Supplementary Figure 14. Morganella morganii, patient 56 and patient 76 (ERIC2-PCR Uncropped gel)
Patient N°56
Patient N°76
UM-3
CB-3
UM-1
BU-12
UM-12
CB-9
CB-12
CB-5
UM-4
CB-4
BU-2
BU-3
CB-1
C+
Comment
This gel was processed in one experiment
This gel presents the samples from two different patients
Each patient was analyzed individually
The result of these analyses is shown on the following 2 slides: Supplementary Figure 15 for patient 56 and Supplementary Figure 16 for patient 76
Legend
UM-n: urinary meatus Day n
CB-n: collector bag Day n
BU-n: bladder urine Day n
C+: positive control

## Slide 20
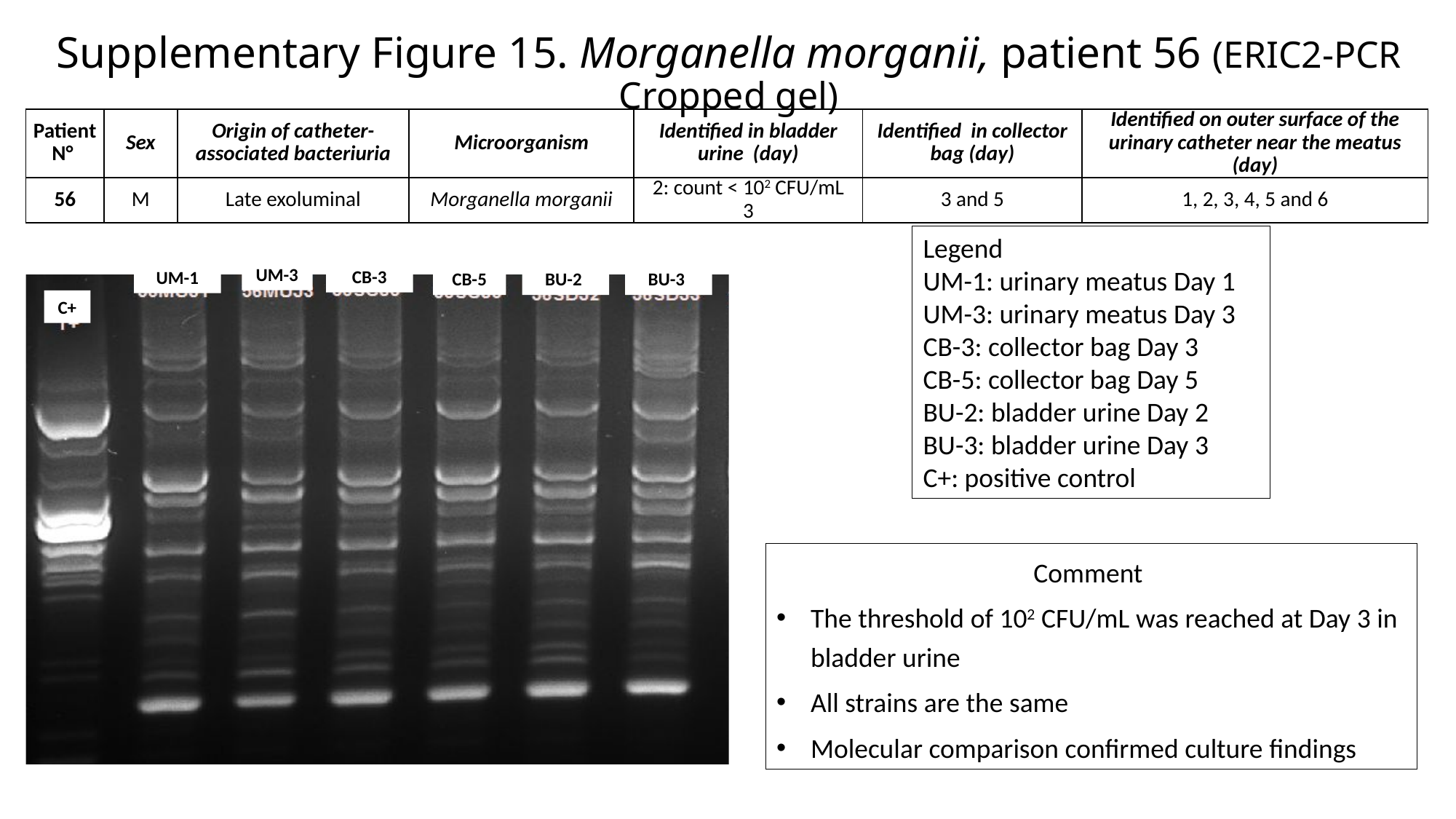

Supplementary Figure 15. Morganella morganii, patient 56 (ERIC2-PCR Cropped gel)
| Patient N° | Sex | Origin of catheter-associated bacteriuria | Microorganism | Identified in bladder urine (day) | Identified in collector bag (day) | Identified on outer surface of the urinary catheter near the meatus (day) |
| --- | --- | --- | --- | --- | --- | --- |
| 56 | M | Late exoluminal | Morganella morganii | 2: count < 102 CFU/mL 3 | 3 and 5 | 1, 2, 3, 4, 5 and 6 |
Legend
UM-1: urinary meatus Day 1
UM-3: urinary meatus Day 3
CB-3: collector bag Day 3
CB-5: collector bag Day 5
BU-2: bladder urine Day 2
BU-3: bladder urine Day 3
C+: positive control
UM-3
CB-3
UM-1
BU-2
BU-3
CB-5
C+
Comment
The threshold of 102 CFU/mL was reached at Day 3 in bladder urine
All strains are the same
Molecular comparison confirmed culture findings

## Slide 21
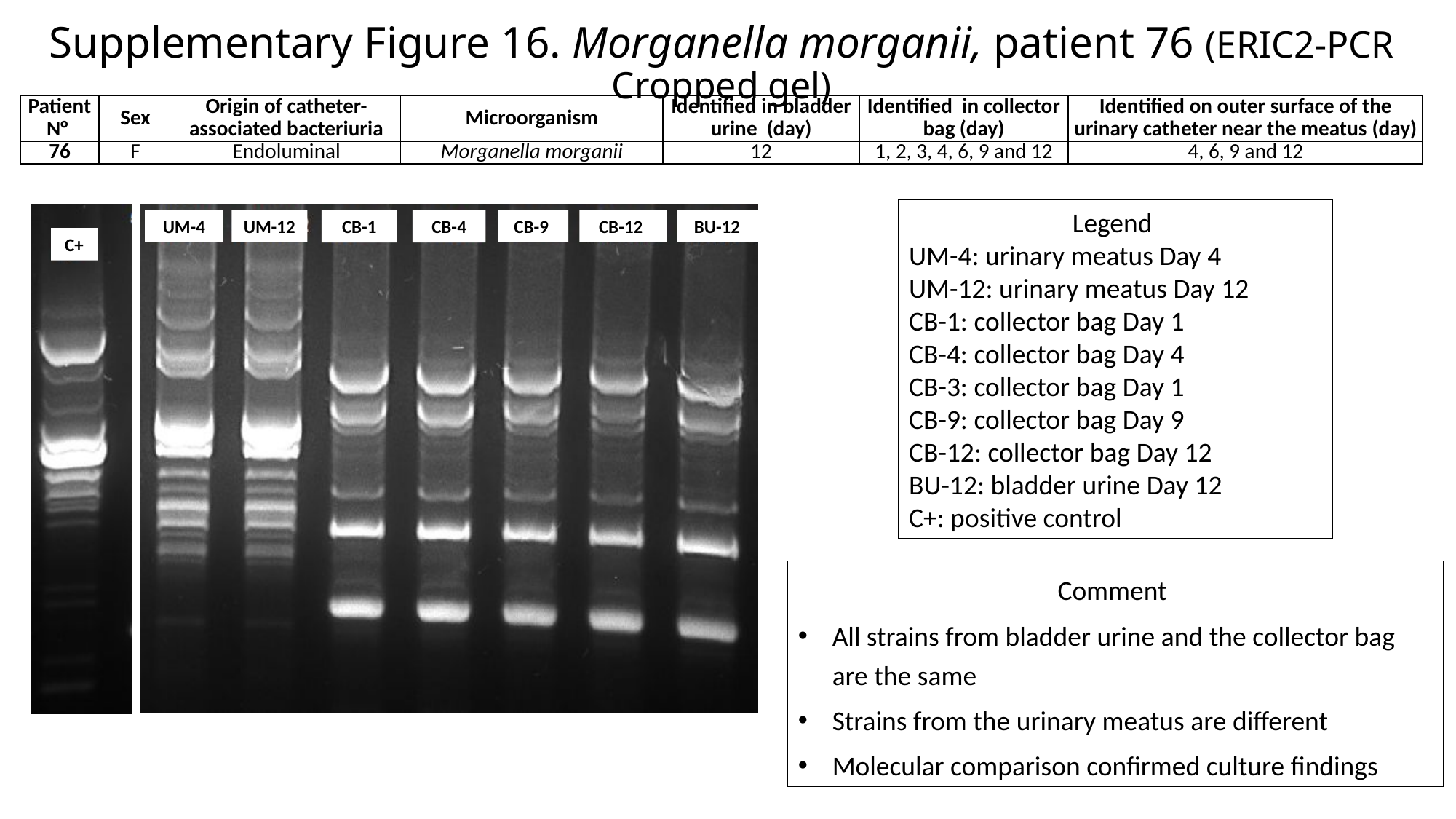

Supplementary Figure 16. Morganella morganii, patient 76 (ERIC2-PCR Cropped gel)
| Patient N° | Sex | Origin of catheter-associated bacteriuria | Microorganism | Identified in bladder urine (day) | Identified in collector bag (day) | Identified on outer surface of the urinary catheter near the meatus (day) |
| --- | --- | --- | --- | --- | --- | --- |
| 76 | F | Endoluminal | Morganella morganii | 12 | 1, 2, 3, 4, 6, 9 and 12 | 4, 6, 9 and 12 |
Legend
UM-4: urinary meatus Day 4
UM-12: urinary meatus Day 12
CB-1: collector bag Day 1
CB-4: collector bag Day 4
CB-3: collector bag Day 1
CB-9: collector bag Day 9
CB-12: collector bag Day 12
BU-12: bladder urine Day 12
C+: positive control
UM-4
UM-12
CB-9
CB-12
BU-12
CB-1
CB-4
C+
Comment
All strains from bladder urine and the collector bag are the same
Strains from the urinary meatus are different
Molecular comparison confirmed culture findings

## Slide 22
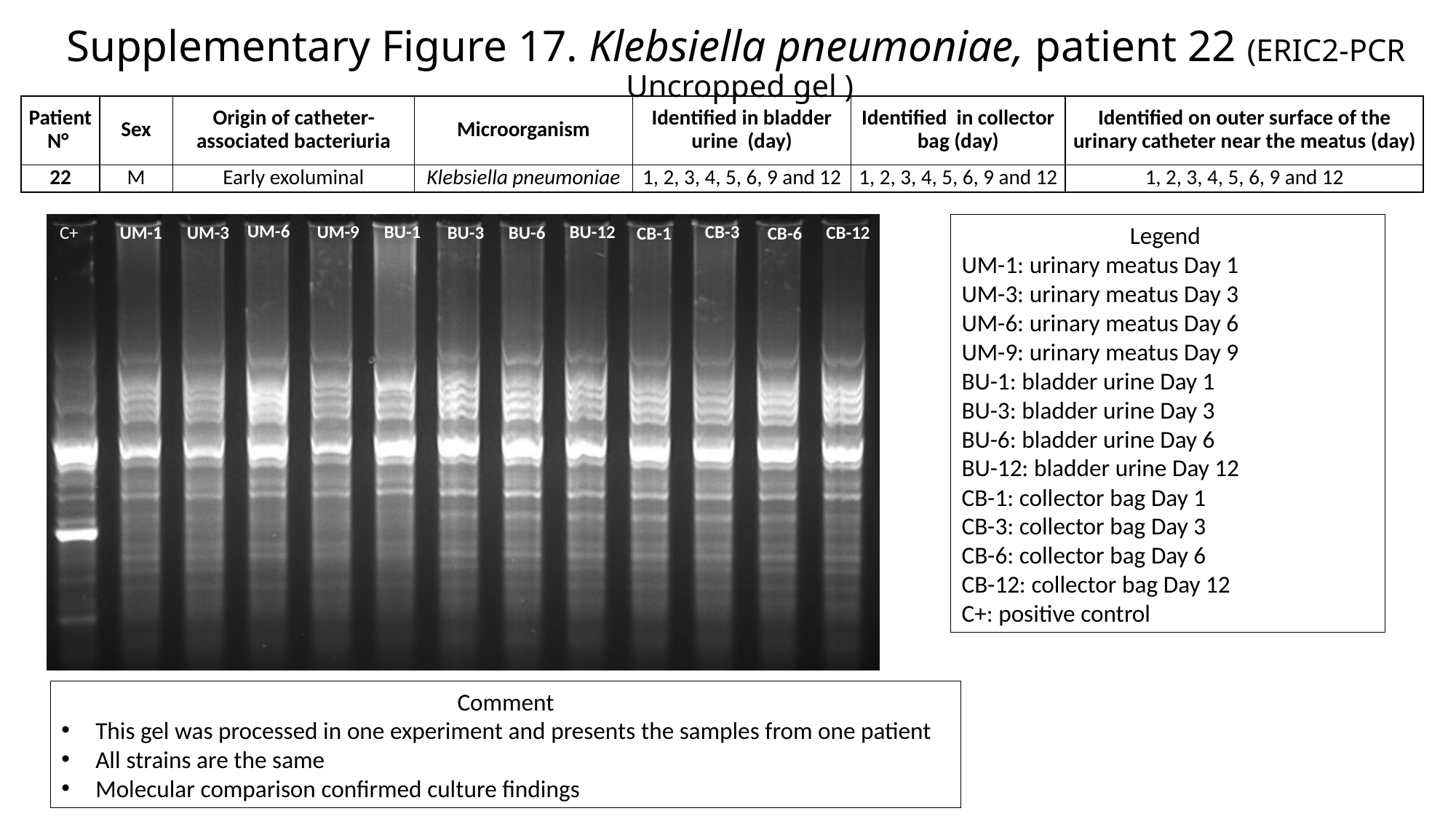

Supplementary Figure 17. Klebsiella pneumoniae, patient 22 (ERIC2-PCR Uncropped gel )
| Patient N° | Sex | Origin of catheter-associated bacteriuria | Microorganism | Identified in bladder urine (day) | Identified in collector bag (day) | Identified on outer surface of the urinary catheter near the meatus (day) |
| --- | --- | --- | --- | --- | --- | --- |
| 22 | M | Early exoluminal | Klebsiella pneumoniae | 1, 2, 3, 4, 5, 6, 9 and 12 | 1, 2, 3, 4, 5, 6, 9 and 12 | 1, 2, 3, 4, 5, 6, 9 and 12 |
UM-6
BU-1
BU-12
UM-9
CB-3
BU-3
BU-6
C+
UM-3
CB-12
UM-1
CB-1
CB-6
Legend
UM-1: urinary meatus Day 1
UM-3: urinary meatus Day 3
UM-6: urinary meatus Day 6
UM-9: urinary meatus Day 9
BU-1: bladder urine Day 1
BU-3: bladder urine Day 3
BU-6: bladder urine Day 6
BU-12: bladder urine Day 12
CB-1: collector bag Day 1
CB-3: collector bag Day 3
CB-6: collector bag Day 6
CB-12: collector bag Day 12
C+: positive control
Comment
This gel was processed in one experiment and presents the samples from one patient
All strains are the same
Molecular comparison confirmed culture findings
